# GARLIC: a bioinformatic toolkit for aetiologically connecting diseases and cell type-specific regulatory maps

**DOI:** 10.1093/hmg/ddw423

**Published:** 2016-12-22

**Authors:** Miloš Nikolić, Argyris Papantonis, Alvaro Rada-Iglesias

**Affiliations:** 1 Center for Molecular Medicine Cologne (CMMC), Robert-Koch-Str. 21, 50931 Cologne, Germany; 2 The Cologne Cluster of Excellence in Cellular Stress Responses in Aging-associated Diseases (CECAD), Joseph-Stelzmann-Straße 26, 50931 Cologne, Germany

## Abstract

Genome-wide association studies (GWAS) have emerged as a powerful tool to uncover the genetic basis of human common diseases, which often show a complex, polygenic and multi-factorial aetiology. These studies have revealed that 70–90% of all single nucleotide polymorphisms (SNPs) associated with common complex diseases do not occur within genes (*i.e.* they are non-coding), making the discovery of disease-causative genetic variants and the elucidation of the underlying pathological mechanisms far from straightforward. Based on emerging evidences suggesting that disease-associated SNPs are frequently found within cell type-specific regulatory sequences, here we present GARLIC (GWAS-based Prediction Toolkit for Connecting Diseases and Cell Types), a user-friendly, multi-purpose software with an associated database and online viewer that, using global maps of *cis*-regulatory elements, can aetiologically connect human diseases with relevant cell types. Additionally, GARLIC can be used to retrieve potential disease-causative genetic variants overlapping regulatory sequences of interest. Overall, GARLIC can satisfy several important needs within the field of medical genetics, thus potentially assisting in the ultimate goal of uncovering the elusive and complex genetic basis of common human disorders.

## Introduction

A major goal in the postgenomic era is to dissect the genetic basis of human disease, which has far-reaching diagnostic and therapeutic implications. Although there has been a remarkable success in uncovering the causative mutations and the relevant genes involved in a number of rare Mendelian diseases (1,2), this has proven considerably more challenging in the case of common and complex human disorders. These disorders are characterized by polygenic and multifactorial aetiology, whereby the combination of certain genetic variants and environmental risk factors contribute to disease susceptibility (3,4). The completion of the human genome project, more than a decade ago, together with the appearance of microarray technology, paved the way for the establishment of Genome-Wide Association Studies (GWAS) (4) as a powerful approach to uncover the genetic basis of common complex diseases. In GWAS, thousands of single nucleotide polymorphisms (SNPs) are typically genotyped in large cohorts of patients and matched controls in order to reveal genetic variants associated with a particular disease or trait. As microarray technology became cheaper and more robust, GWAS bloomed and genetic association data are now available for hundreds of human diseases and traits (*e.g.* GWAS Catalog (https://www.ebi.ac.uk/gwas/). However, the initial excitement regarding GWAS was somewhat dampened by the recurrent observation that most (70–90%) disease-associated genetic variation lie outside genes, within the vast non-coding fraction of the human genome (5,6). Consequently, the molecular and pathological consequences of many of the uncovered genetic variants remain unknown (7–9).

As GWAS became a regular tool in medical genetics, the genomics field was revolutionized by the emergence of next generation sequencing (NGS). Among its many applications, NGS has been instrumental in the functional annotation of the human genome. In this regard, epigenomic profiling and the use of chromatin signatures revealed that the human genome is densely populated by *cis*-regulatory elements (CREs; *i.e*. promoters, enhancers, insulators). Amongst them, enhancers, which can control gene expression in a distance- and orientation-independent manner, seem to be particularly abundant and cell type-specific. Using epigenomic approaches such as ChIP-seq, DNAse-seq or ATAC-seq, regulatory maps have now been generated in hundreds of different human cell types and tissues, mostly as part of large international efforts such as The Encyclopedia of DNA Elements (ENCODE) (5), the Roadmaps Epigenomics (10) or the BLUEPRINT (11) consortia. Importantly, global analysis of GWAS data and CRE maps suggests that a significant fraction of all non-coding genetic variants associated with common complex human diseases lie within putative *cis*-regulatory elements, especially within enhancers (6). Moreover, SNPs associated with a particular disease were frequently overrepresented within CREs present in cell types or tissues thought to be relevant for that disease. Consequently, it has been hypothesized that SNPs occurring within CREs might alter the regulatory properties of these sequences and lead to quantitative changes in gene expression with potentially pathological consequences (8,12–16).

In theory, the combination of GWAS information and CRE maps from different human cell types and tissues should streamline the identification of causative genetic variants for common complex diseases (8,17,18). However, using candidate-based approaches, this has been so far accomplished in only a handful of SNPs and CREs, which have nevertheless led to the discovery of novel genes and pathways involved in relevant human disorders (7,16,19–23). There are probably multiple reasons explaining this moderate success in uncovering disease causative non-coding variants: (i) SNPs reported in GWAS should be considered as mere markers of larger human haplotypes. Thus, they are not necessarily causative and, instead, they might be in linkage-disequilibrium (LD) with the true causative variants (8); (ii) CREs, especially enhancers, are highly dynamic and cell-type specific. Thus, regulatory and pathological effects of disease-causative SNPs might only be revealed if investigated in the relevant human cell types or tissues (6,24); (iii) Most attempts to investigate the pathological consequences of non-coding SNPs have focused on cell types/tissues that are considered relevant for a particular disease based on previous knowledge (6,25). However, the repertoire of cell-types/tissues that are important for particular common complex diseases might not be completely understood in some cases; (iv) GWAS and epigenomic data are not readily accessible to the average user, typically requiring some computational skills in order to formulate testable hypothesis regarding the genetic basis of human disease. Here we present a novel software and associated database, named GARLIC (GWAS-Based Prediction Toolkit for Connecting Diseases and Cell Types), which aims at minimizing all the previously mentioned issues in order to provide a user-oriented, user-friendly and systematic approach to facilitate the genetic dissection of common and complex human disorders.

## Results

### The GARLIC database

**Figure 1.**
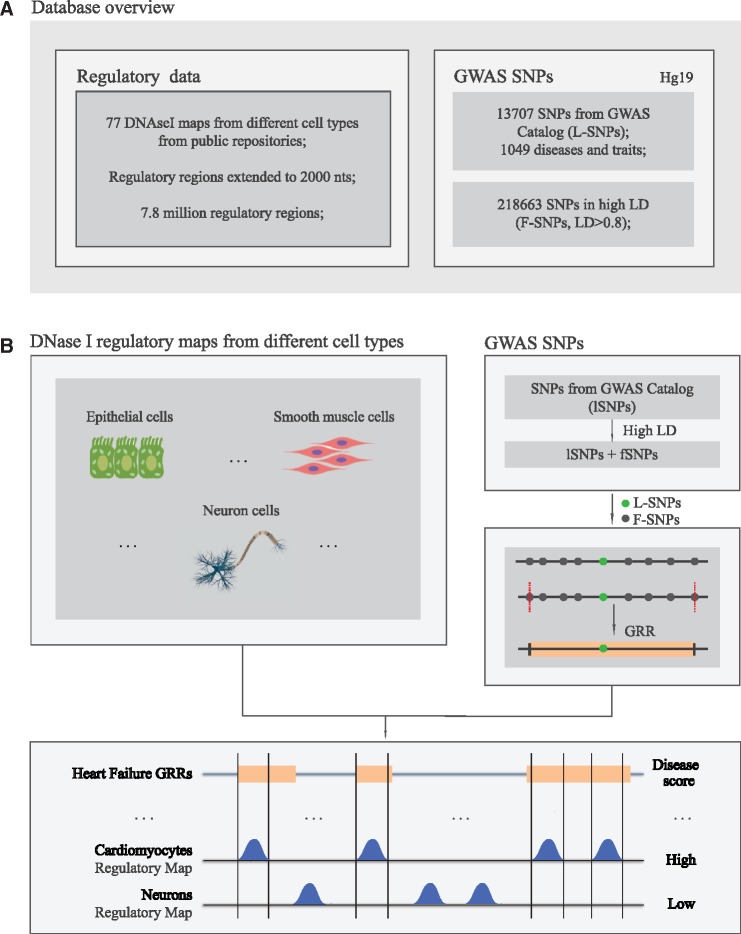
Overview of the GARLIC rationale and the included datasets. (**A**) SNPs causally involved in common complex diseases are predicted to occur within CRE present in disease-relevant cell types. These SNPs can alter the regulatory properties of CRE, which can lead to quantitative changes in gene expression and increased disease susceptibility. (**B**) GARLIC major underlying hypothesis is that the regulatory maps from the cell types or tissues most relevant for a given disease should be preferentially enriched in disease-associated SNPs in comparison with non-relevant cell types.

A central component of GARLIC is a local database (DB) designed to store and combine different data sets: (i) SNPs reported in GWAS as associated with a list of 1049 different diseases (Lead SNPs (L-SNPs), total = 13707) and those SNPs that are in high LD (*r*^2 ^>^ ^0.8) with the L-SNPs (Follower SNPs (F-SNPs, total = 218663), resulting in a total of 232370 SNPs; (ii) CRE maps generated by DNase-seq in 77 different human cell types and tissues (from the ENCODE (5), Roadmap Epigenomics (10) and BLUEPRINT (11) repositories) (Supplementary Material, Table S1). The user can also add to the DB novel CRE maps generated in any human cell type or tissue of interest, which can in principle be generated by several alternative methods (DNAse-seq, ChIP-seq, ATAC-seq, CAGE) or combinations thereof. Similarly, the user can also add lists of SNPs identified as associated with a disease or trait in novel GWAS studies. An overview of the integrated datasets used in the DB is shown in Figure 1A (complete DB schema is included as Supplementary Material, Fig. S1).

The GARLIC DB can be fully accessed using MySQL querying techniques. Moreover, basic DB query functions are provided through several wrap-up shell scripts requiring no prior programming knowledge, thus making the DB accessible to a broader audience.

### Predicting aetiological connections between diseases and cell types/tissues

The GARLIC toolkit includes a novel method to aetiologically connect human diseases and traits with different cell types or tissues based on the overlap between disease genomic risk-regions (GRRs) and CRE maps (Fig. 1B; see Methods for details). It is worth mentioning that the GARLIC method does not require GWAS SNPs (L-SNPs or F-SNPs) to be directly located within CREs. In addition, we speculated that, by combining a large number of diseases/traits and CRE maps it should be possible not only to confirm previously known aetiological connections between diseases and cell types/tissue, but also to predict some novel and unexpected ones.

**Figure 2.**
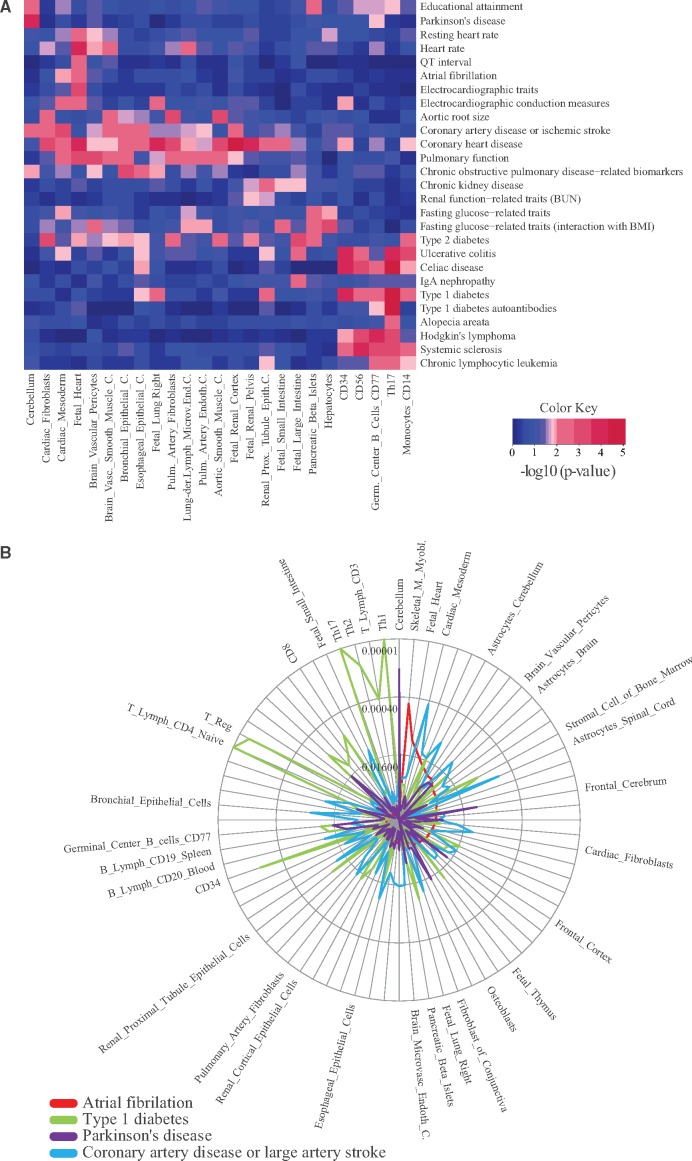
GARLIC can be used to aetiologically connect human complex diseases and cell type-specific CRE maps. (**A**) GARLIC results obtained for a selected subset of diseases (rows) and CRE maps (columns) are shown as a heat map. The statistical connection between each disease and cell type is color coded according to GARLIC *P*-values, with the most and least significant connections represented in red and blue, respectively. (**B**) Radial plot summarizing the statistical connection between four selected diseases (indicated in the bottom left corner) and all cell types included in the GARLIC DB. The name of only a subset of all the investigated cell types is shown. Peaks closer to the outer border of the radial plot represent more significant connections, while those closer to the center are the least significant ones.

In order to test our method, we focused on diseases included in the DB that have seven or more GRRs. This ensured sufficiently large sample sizes, and was thus compatible with the random sampling procedure described in the Methods section. After applying this criterion, we were able to test 510 out of the 1049 diseases included in the GARLIC DB against the 77 CRE maps from the different human cell types and tissues. To illustrate our method’s performance, the aetiological connections between a selected subset of 27 diseases and 25 CRE maps are depicted in Figure 2A. The results of testing all CRE maps against 167 diseases for which a statistically-significant connection (*P*≤0.01) was found with at least one CRE map are presented in Supplementary Material, Fig. S2. From a global perspective, it is obvious that some diseases/traits showed highly significant connections with a large number of cell types/tissues, probably reflecting their complex and pleiotropic genetic basis (*e.g*. height, platelet counts). In contrast, other diseases were almost exclusively connected to just a single CRE map (*e.g*. Parkinson’s disease with cerebellum CRE map). Moreover, distinct clusters connecting related group of diseases and cell types could also be visualized (*e.g*. autoimmune diseases with immune system-related cell types) (Supplementary Material, Fig. S2). Overall, there were many statistically significant connections in agreement with the current understanding of various human diseases and traits and we discuss some of these below.

We anticipate that GARLIC users might be interested in accessing the previous aetiological connections from two different standpoints: users might be particularly interested in the diseases showing a statistically significant connection with a given cell type/tissue or, alternatively, might want to predict which cell types/tissues are more relevant for a given disease/trait. Using simple command-line tools or a dedicated, user-friendly online interface, the GARLIC Viewer (http://bifacility.uni-koeln.de/GARLIC/viewer.php), GARLIC allows the user to retrieve these connections for any given cell type/tissue or disease/trait of interest. To illustrate the former, we show the results for four different cell types/tissues: fetal heart (full results from this regulatory map are listed in Supplementary Material, Table S2 to exemplify the output generated by GARLIC), T-lymphocytes (CD3+), astrocytes, and fetal renal pelvis (Supplementary Material, Fig. S3). The results obtained for other cell types are fully accessible through the GARLIC Viewer. In the case of fetal heart and T-lymphocytes, highly significant connections mostly with expected diseases and traits were observed (*i.e*. fetal heart: *heart rate, QT interval, electrocardiographic traits, atrial fibrillation, coronary heart disease*; T-lymphocytes: autoimmune disorders such as *Celiac disease*, *Psoriasis*, *Type I diabetes* and *atopic dermatitis*). In contrast, astrocytes did not associate with neurological disorders and instead showed significant connections with non-neural diseases, including *intracranial aneurysm* and *ischaemic stroke*. This suggests that astrocytes might play a causative (yet largely unexplored) role in cerebrovascular disease via non-coding mutations occurring within astrocyte CREs. Similarly, when the CRE map from the fetal renal pelvis (the broadened top part of the ureter into which the kidney tubules drain) was considered, statistically significant connections were observed not only to kidney-related diseases and traits, but also to other somehow unexpected human conditions, such as *metabolic syndrome*. Interestingly, this complex metabolic disorder also displayed a strong connection with immune system cell types, indicating that uncovering the genetic basis of this disease might require considering not only tissues involved in metabolic regulation (*e.g*. liver, pancreas, fat tissue), but also unexpected contributors, such as the kidney and the immune system.

On the other hand, to illustrate the performance of GARLIC when focusing on particular diseases, results for four common complex diseases (*i.e*. *coronary artery disease or large artery stroke*, *Type I diabetes*, *Parkinson’s disease* and *atrial fibrillation*) are shown in Figure 2B. These diseases were selected based on the varying number of cell types/tissues with which they seem to be connected. *Coronary artery disease* displayed moderate connection with a large number of cell types/tissue, suggesting a complex and pleiotropic aetiology. *Type I diabetes* also strongly associated with a large number of cell types, but in this case, most of them were related to the immune system. In contrast, *Parkinson’s disease* and *atrial fibrillation* were considerably more specific, as they showed highly significant connections to only a few cell types [*i.e*. Parkinson’s disease: cerebellum (*P* = 7×10^−^^5^) and frontal cerebrum (*P* = 6.7×10^−^^3^); atrial fibrillation: skeletal muscle (*P* = 6×10^−^^4^), fetal heart (*P* = 6.1×10^−^^3^), cardiac mesoderm (*P* = 1.07×10^−^^2^)].

Overall, these results illustrate how GARLIC can be used to systematically link human diseases/traits and cell types/tissues, which in some cases might lead to unexpected yet potentially relevant aetiological insights. Importantly, users can also incorporate novel CRE maps or GWAS datasets into GARLIC that can be then analysed with respect to all other datasets already present in the DB.

### Combinatorial procedure to identify the cell type pairs with an increased aetiological contribution to human disease

Despite the statistically significant aetiological connections observed between many human diseases/traits and specific cell types/tissues, there were still a large number of diseases for which strong aetiological associations could not be made (*e.g.* 343 diseases/traits did not achieve *P*-value < 0.01). Due to the polygenic and the multifactorial aetiology of most common human diseases, it is conceivable that certain disorders will not display strong aetiological connections with any individual cell type or tissue. Moreover, it is also possible that CRE maps are currently not available for the cell types/tissues that might be more relevant for certain human diseases/traits. Alternatively, multiple cell types/tissues may all moderately contribute to such diseases through specific CREs. To address this possibility without using a brute force approach, we implemented a software feature that identifies combinations of CRE maps from different cell types/tissues that achieve more significant results for a given disease/trait than when regulatory maps are tested individually (Supplementary Material, Fig. S4A; see Methods). To exemplify the potential of this GARLIC feature, we prioritized diseases/traits for which our previous individual CRE maps did not find strong aetiological connections (*P*-value > 0.01). Among these diseases/traits, we tested 95 (out of 343) and found seven of them having at least one CRE pairwise map combination that yielded a lower *P*-value in comparison to the results obtained when the two corresponding CRE maps were tested separately (Supplementary Material, Fig. S4B). Amongst the identified CRE map combinations, those that yielded a lower *P*-value than all individually tested CRE maps were particularly interesting, as they illustrate the potential of our combinatorial procedure to discover aetiological connections that would be missed otherwise. For example, for the trait *Dietary macronutrient intake*, the merged CRE map for Choroid plexus epithelial cells and Frontal Cortex achieved a *P*-value (0.019) that was lower than either of these two maps when tested separately (*P*-value = 0.168) or, more importantly, than any of the individually tested CRE maps (*P*-value = 0.045). These results suggest that genetic variants located within CREs present in different parts of the brain might contribute to the control of nutrient intake, which is believed to have an important neural component (26). Similarly, for *Response to serotonin reuptake inhibitors in major depressive disorder*, a significant association was obtained for the combination of Hepatic Stellate Cells with Renal Cortical Epithelial Cells, suggesting that the response to these drugs might depend on how they are metabolized in the liver and the kidney.

### Extracting disease-associated SNPs overlapping CREs or loci of interest

**Figure 3.**
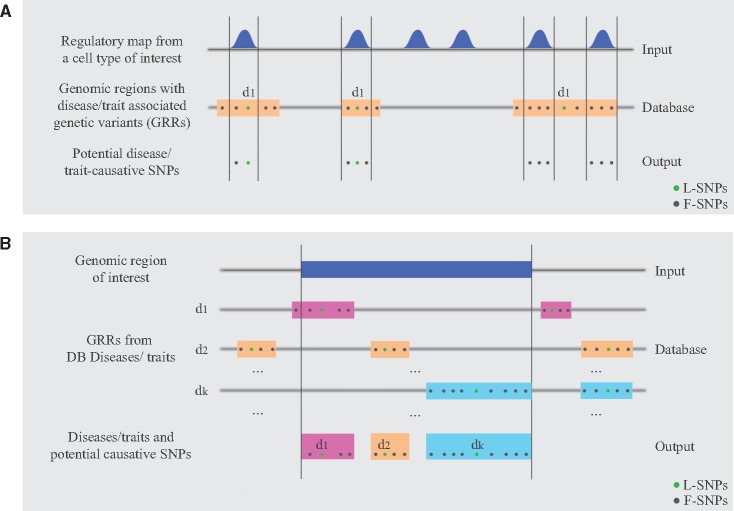
Identification of SNPs located within CREs of interest. (**A**) SNPs overlapping CREs and their associated-diseases can be retrieved using as input either regulatory maps or (**B**) a single locus of interest.

GARLIC aetiologically links cell types/tissues and diseases/traits based on the overlaps between CREs and GRRs, which does not require that the actual GWAS SNPs (L-SNPs and F-SNPs) occur directly within CREs. Nevertheless, once cell types/tissues and diseases/traits are aetiologically connected, GARLIC enables the identification of all GWAS SNPs (both L-SNPs and F-SNPs) associated with a disease/trait of interest and overlapping CREs in a given cell type/tissue (Fig. 3A). Using simple command-line tools (see Online user manual, https://github.com/mnikoli/GARLIC) or the GARLIC viewer, this can provide the user with a limited list of SNPs that can be prioritized for downstream *in silico* (27–30) and/or experimental analyses to evaluate their potential role as disease-causative SNPs. CRE maps already included in the GARLIC DB or any other CRE map generated through different techniques, such as DNAse-seq, ATAC-seq or ChIP-seq, may be used. For example, when considering congenital heart malformation GWAS data and the fetal heart CRE map as inputs, we found nine out of 55 SNPs associated with this malformation within two different CREs (Table 1). Eight of these SNPs occurred within a single CRE (chr4: 140797201-140796912; hg19) located within an intron of *MALM3*. Interestingly, *MALM3* is a coactivator of the NOTCH signalling pathway, which is known to play a crucial role in cardiac development and heart congenital diseases (31).

**Table 1.** SNPs associated with congenital heart malformation overlapping CREs from Fetal heart

Name	Chr	Position	Overlapping Region
rs12725053	chr1	118939846	118938659 – 118940963
rs11100326	chr4	140797683	140796912 – 140800330
rs1455480	chr4	140800330	140796912 – 140800330
rs150260800	chr4	140798452	140796912 – 140800330
rs17368602	chr4	140800012	140796912 – 140800330
rs4863518	chr4	140799733	140796912 – 140800330
rs4863519	chr4	140799749	140796912 – 140800330
rs4863699	chr4	140799855	140796912 – 140800330
rs6834463	chr4	140797201	140796912 – 140800330

Conversely, instead of using CRE maps, users can provide genomic coordinates for a single locus of interest as input [*e.g*. gene, lincRNA, topological domain (TAD)] for which pathological connections want to be investigated (Fig. 3B). GARLIC can then be used to retrieve all diseases for which L-SNPs or F-SNPs occur within the locus of interest, together with a detailed list of all those SNPs. As an example, we looked into the *TBX5* gene locus, which encodes a transcription factor considered as a master regulator of heart development (32). The *TBX5* genomic coordinates were extended by ±1 Kb and used as input for the identification of 20 disease-associated SNPs. Interestingly, despite the large number (>1000) of diseases and traits included in our DB, all identified SNPs were associated with heart-related functions: *PR interval*, *QRS duration* and *electrocardiographic traits* (Table 2). A detailed description of all commands and calling parameters required for the two strategies described above is provided in the Online user manual.

**Table 2.** SNPs associated with different diseases and traits located within the *TBX5* locus

Name	Chr	Position	SNP_type	Associated_disease(s)
rs1895585	chr12	114802138	L	PR interval
rs3825214	chr12	114795443	L	Electrocardiographic traits
rs883079	chr12	114793240	L	QRS duration
rs10507248	chr12	114797093	F	PR interval; QRS duration
rs10744823	chr12	114798082	F	Electrocardiographic traits
rs10744824	chr12	114808638	F	Electrocardiographic traits
rs12367410	chr12	114796688	F	Electrocardiographic traits
rs148020424	chr12	114805057	F	PR interval; QRS duration
rs1895582	chr12	114807035	F	PR interval; QRS duration
rs1895583	chr12	114806885	F	PR interval
rs1946293	chr12	114802760	F	PR interval; QRS duration
rs1946295	chr12	114802361	F	PR interval; QRS duration
rs2113433	chr12	114794057	F	Electrocardiographic traits
rs3825215	chr12	114804898	F	PR interval; QRS duration
rs4767237	chr12	114800813	F	PR interval; QRS duration
rs6489956	chr12	114792236	F	Electrocardiographic traits
rs7135659	chr12	114801772	F	PR interval; QRS duration
rs7312625	chr12	114799974	F	PR interval; QRS duration
rs7316919	chr12	114791455	F	Electrocardiographic traits
rs7955405	chr12	114797306	F	PR interval; QRS duration

Finally, although GARLIC was originally designed to investigate the aetiological connection between diseases and cell types based on CRE maps, it can also make such connections using other types of regulatory maps (as far as they are provided in BED format) potentially enriched in disease-associated genetic variants. To illustrate its broad applicability, we used GARLIC with regulatory maps from recursive splicing sites (RSSs) in two different cell types, human umbilical vein endothelial cells and neural progenitors. Recursive splicing is thought to mediate the excision of long human introns (33,34) and it is conceivable that SNPs mutating such sites can interfere with proper mRNA maturation. Due to the low number of recursive splice sites per cell type, we applied our method without any prior removal of disease-associated GRRs. Results connected variation at endothelial cell RSSs mostly to *smoking* and *chronic obstructive pulmonary disease*, which, interestingly, are known to become aetiologically linked due to initial lesions occurring in the lung endothelium (35). Likewise, variation at neuronal progenitor RSSs associated to diseases like *migraine* and *Parkinson’s*, or to *cocaine and alcohol consumption* (Supplementary Material, Fig. S5), which are all diseases and traits with a well-known neurological component.

## Discussion

GWAS have emerged as a powerful and broadly-used strategy to uncover the genetic basis of common and complex diseases. As a result, thousands of genetic variants have now been associated with various human disorders and numbers keep increasing at a remarkable pace. However, as GWAS data accumulate in dedicated databases, relatively little progress has been made regarding the molecular and pathological characterization of most of the uncovered genetic variants. Therefore, there is a major need to identify disease-causative genetic variants, as this can dramatically increase the diagnostic and therapeutic impact of existing and forthcoming GWAS.

A major reason that explains the difficulty in moving from GWAS to the identification of disease-causative genetic variants is that the vast majority of disease-associated genetic variation occurs within human non-coding sequences (6). Based on accumulating evidences, we and others have hypothesized that a significant fraction of disease-causative non-coding genetic variants can disrupt CREs, such as enhancers, and therefore lead to pathological changes in gene expression (8,12,13). Since many enhancers are cell-type specific, the impact of genetic variants of interest might only be revealed if investigated in the relevant cell type/tissue (6,36). With these ideas in mind, we developed GARLIC, a multipurpose and user-oriented toolkit that enables to aetiologically connect human diseases with relevant cell types/tissues in a systematic and unbiased manner. To test GARLIC performance, we first analysed a large cohort of GWAS datasets and CRE maps (510 GWAS and 77 CRE maps). In most previous studies (6,25,37) in which CRE maps were used to link diseases and cell types, the *in silico* tools developed were not made publicly-available and, in addition, major emphasis was given to finding previously known aetiological connections. In other cases in which the implemented tools were made available (36,38), the user has to retrieve and provide the relevant GWAS datasets and CRE maps, which requires some background in genomics and basic computational skills. To overcome these limitations, GARLIC allows users to not only analyse the link between any GWAS dataset and CRE map of interest, but also, to explore globally and neutrally all the data included in our database. Similar to previous studies (25,37), we found strong statistical support for already established or expected aetiological connections between certain disorders and cell types (*e.g*. autoimmune disorders and immune-system cell types), thus validating the performance of our approach. However, we also observed numerous novel and unexpected aetiological links that might merit further experimental analysis, as illustrated for example by the potential involvement of astrocytes in cerebrovascular disease.

The prediction of disease-causative SNPs is neither the primary nor the major feature of GARLIC, which is especially designed to make statistical predictions regarding the aetiological connections between diseases and human cell types/tissues. To make these connections, GARLIC relies on the overlap between the GRRs of human diseases and the CRE maps of different cell types/tissues, without requiring GWAS SNPs (*i.e.* L-SNP or F-SNPs) to be actually located within CREs. Using this strategy, we tried to minimize the possibility that disease-causative genetic variants might not be part of the considered GWAS SNPs, which can frequently occur due to the disease contribution of rare genetic variants and/or variants showing lower LD (*i.e. r*^2 ^<^ ^0.8) with the L-SNPs than required by GARLIC. Nevertheless, once diseases and cell types are aetiologically linked, GARLIC can be used to extract all disease-associated SNPs overlapping CREs within the cell type/tissue of interest. These SNPs represent potential disease-causative genetic variants that can be prioritized for further analysis via other *in silico* tools designed *ad hoc* for the prediction of non-coding regulatory variants (27–30), followed by experimental validation. Additionally, although we focused on CREs as the key non-coding sequences harbouring disease relevant genetic variations, using recursive splicing sites from two different cell types as an example (33,34), we show that GARLIC can use as input other lists of genomic loci with potential medical relevance (*e.g*. lincRNAs, splicing regulatory sequences).

The GARLIC toolkit and its associated DB offer a systematic and user-friendly approach to investigate the aetiological and genetic basis of common and complex human disorders. We believe that the variety of implemented features and its usability render GARLIC accessible for a broad scientific audience. Finally, whole-genome NGS approaches, which are expected to be superior to GWAS in uncovering the disease-causative genetic variants, are now used to investigate the genetic basis of an increasing number of complex diseases (39). In parallel, recent methodological advances in genomics, such as ATAC-seq or single-cell ChIP-seq (40,41) will enable the generation of CRE maps from hitherto inaccessible human cell types/tissues, including patient material. Therefore, we anticipate that there will be an increasing need to combine genetic information with the continuously improved functional annotation of the human genome. Hence, we believe that, with further optimization and frequent updates, GARLIC will be an important asset in facing these upcoming challenges.

## Materials and Methods

### GARLIC database and data preprocessing

**Figure 4.**
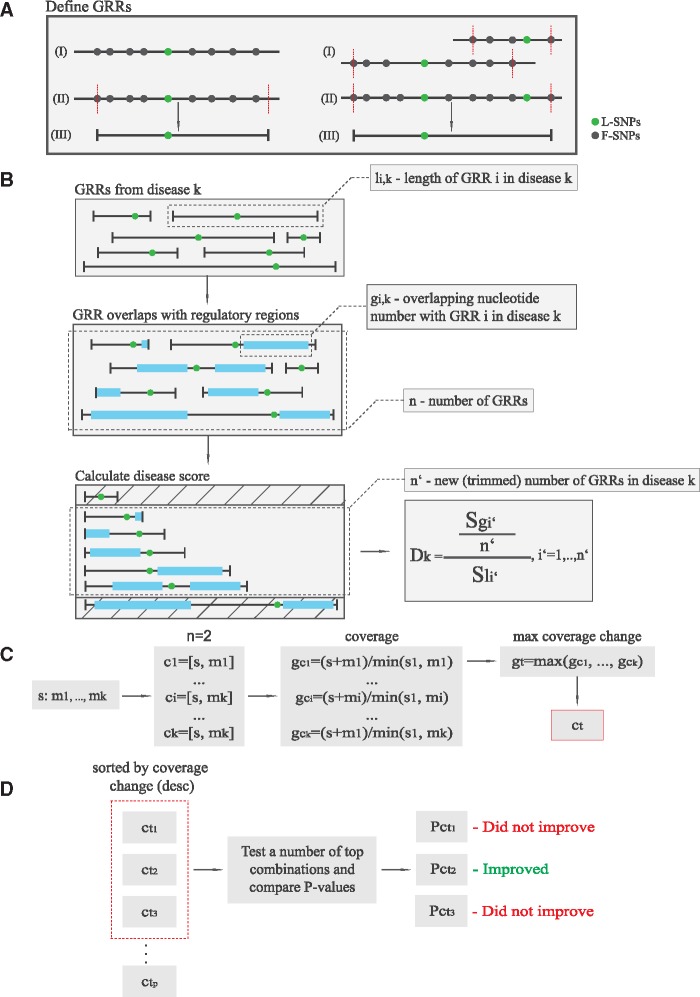
Graphical overview of GARLIC procedures. (**A**) As part of the data preprocessing, unique sets of GRRs for each disease and trait are generated. This is illustrated when either a single (Left) or multiple L-SNPs in LD (Right) are considered. (**B**) Each GRR gets assigned a GRR length and number of overlapping bp with a given regulatory map. The 20% shortest and longest GRRs are excluded and the remaining 60% of GRRs are used to calculate disease scores from which empirical *P*-values can be derived using the random sampling procedure. (**C**) Each seed map gets assigned a set of “complementary” regulatory maps, which are then used to generate “combined” maps. The number of overlapping bp with a given disease or trait of interest is calculated for each combination and only the one with the highest increase in coverage is kept for the next step of the procedure. (**D**) Seed combinations with the highest increase in bp coverage can then be tested for statistical connection with human diseases/traits using the same method employed with individual CRE maps. The number of seed combinations to be tested can be determined with input parameters based on coverage increase.

We designed and implemented a MySQL DB as an integral part of the GARLIC software to accommodate all available SNP data from the GWAS Catalog (https://www.ebi.ac.uk/gwas/) [accessed on 15.06.2015]. Considering that SNPs reported in GWAS, which we will refer to as lead SNPs (L-SNPs), are not necessarily disease-causative, additional SNPs (follower SNPs (F-SNPs)) having an *r*^2^ > =0.8 with the L-SNPs were identified using HaploReg tool (http://archive.broadinstitute.org/mammals/haploreg/haploreg_v2.php; date last accessed June 15, 2015). 46 entries from the GWAS Study named “A genome-wide search for common SNP x SNP interactions on the risk of venous thrombosis” representing epistatic interactions between pairs of SNPs were discarded and not included in GARLIC DB. Next, genomic risk-regions (GRRs) for each L-SNP were defined by the position of the corresponding F-SNPs located furthest upstream and downstream from the L-SNP (Fig. 4A, Left). GWAS studies are not always performed with the same genotyping platforms and thus, different L-SNPs can be in principle used as markers of the same common human haplotypes, which can introduce dependencies among the data. Therefore, for each GRR with multiple L-SNPs in LD associated with the same disease, all those L-SNPs were considered as F-SNPs (ex-L-SNPs) except one that was kept as a name tag for the GRR, while all F-SNPs from ex-L-SNPs were assigned to be F-SNPs of the remaining L-SNP (Fig. 4A, Right). Additionally, all GRRs were assigned to one of five different sized-bins according to their GRR length *l*: [1–1000 bp], [1000–10000 bp], [10000–25000 bp], [25000–55000 bp], [55000–Inf bp), to ensure that randomly-sampled GRRs from the GARLIC DB were of similar size to those tested when investigating the aetiological connection between cell types/tissues and diseases/traits (as described below). GRRs with less than 1000 bp in length were extended to 1000 bp to minimize potential underestimation of the disease scores. In total, we stored GWAS data for 1049 common and complex diseases and traits, which resulted in 15101 GRRs including 13707 L-SNPs and 218663 F-SNPs.

DNAse-seq data (*i.e.* DNAseI hypersensitivity sites (DHS) sequencing) generated by the ENCODE, Roadmap Epigenomics and BLUEPRINT (5,10,11) were gathered for 77 healthy human cell types/tissues either directly from the consortia databases or the CistromeFinder database (http://cistrome.org/finder/; date last accessed November 20, 2016). DNAse-seq peaks were identified using MACS2 or Hotspot (42,43). For MACS2, a false-discovery rate (FDR) (44) cutoff of 0.01 was applied, while for Hotspot, different FDR cutoffs were used by the different consortia (0.01 for Roadmap Epigenomics and ENCODE; 0.05 for BLUEPRINT). In order to define CREs, the DNAse-peaks were equally extended in both the 5' and 3' directions to a final size of 2 kbp and overlapping intervals were merged using BEDtools (45). A detailed description of all the CRE maps used by GARLIC is provided in Supplementary Material, Table S1.

### A random sampling procedure for determining statistically-significant aetiological connections between diseases/traits and cell type/tissues

Using the data stored in GARLIC DB, a scoring method based on the abundance of CREs within previously described GRRs was developed (illustrated in Fig. 4B) to determine aetiological connections between diseases/traits and cell types/tissues, as follows: Firstly, given the input regulatory map, let

$$l_{i}{,}_{k},{\text{ and }g}_{i}{,}_{k},\;\;\;\;\;i=1,\ldots ,n,k=1,\ldots ,m,$$

represent the length in bps and the number of overlapping nucleotides of the *i*^th^ GRR associated with disease/trait *k*, respectively; *n* the number of independent GRRs associated with disease/trait *k* and *m* total number of diseases or traits from GARLIC DB that will be tested for a regulatory map. Secondly, let

$$g_{k}=\left[g_{1},\;\ldots ,\;g_{n}\right],k=1,\ldots ,m$$

be a vector of GRRs associated with disease or trait *k*, sorted in ascending order by GRR lengths l_*i*__,__*k*_. GRRs displayed a great variability in their length, ranging from one bp up to hundreds of Kb. Extremely large GRRs are likely to display high overlaps with CREs regardless of the input regulatory map, potentially inflating the aetiological connections of certain diseases or traits. Similarly, a large number of small GRR within a particular disease/trait may also affect the overall disease score, as the score is based on the arithmetic mean. Therefore, 20% of GRRs from the beginning and end of the sorted **g_*k*_** vector were excluded and the remaining 60% of GRRs were further used in this procedure. Finally, the remaining GRR elements g_*i’,k*_ and their corresponding lengths l_*i’,k*_, were used to calculate *D_k_* – the observed score for the disease or trait k, defined as

$$D_{k}=\frac{\frac{\sum_{i^{\prime }}g_{i^{\prime },k}}{n^{\prime }}}{\sum_{i^{\prime }}l_{i^{\prime },k}},\;i\prime =[0.2*n]+1,\;\ldots ,[0.8*n],n\prime -\mathrm{number}\mathrm{of}\mathrm{GRRs}\mathrm{after}\mathrm{trimming},$$

representing the mean fraction of the covered (overlapping) regions, normalized by the total length of the remaining GRRs. The relevance of the observed score *D_k_* in a given regulatory map was tested by calculating empirical *P-*values, as described in (46). Briefly, for each disease and trait k, a number of GRRs corresponding to the number and size of GRRs in disease or trait k were sampled without replacement 10^5^ times from the whole GARLIC DB. In each iteration, the simulated disease scores were compared to the observed one. The number of occurrences in which a simulated score was higher than the observed one was counted and divided by the total number of iterations increased by one. Finally, calculated empirical *P*-values were corrected using the Benjamini-Hochberg procedure (44) and both values were reported as part of the output. Since the given precision of 10^−^^5^ was not sufficient to provide the actual empirical *P*-values of some diseases and traits in most of the tested regulatory maps, corrected *P*-values represent the worst case scenario with respect to the potential rate of false positives. The described procedure is parallelized and testing all 510 diseases and traits for each regulatory map takes around 30 minutes on a PC with Intel I7 CPU using 8 threads. All these results can be accessed online using the GARLIC viewer (http://bifacility.uni-koeln.de/GARLIC/viewer.php).

### Combinatorial procedure for identifying groups of cell types with increased combined aetiological contribution to complex diseases or traits

This method heavily relies on the previously described procedure, as it uses the CRE maps that were already analysed and stored in the GARLIC DB. Given a disease or trait as input, CRE maps from various cell types are combined in order to increase the likelihood of aetiological contribution of the combined CRE map with a given disease or trait, compared to the association of individual CRE maps when tested separately (Fig. 3A). First, for each CRE map in the DB, the coverage of the GRRs associated with a given disease or trait was calculated. Next, regulatory maps having a coverage in the s^th^ percentile were kept and referred to as seeds or seed maps, as they are “fixed” in the sense that they always occupy the first position of all possible seed combinations, while other combination members are then added to form pairs, triplets, etc. For parameter s, we used 0.6. Next, for each seed map, regulatory maps that overlap GRRs or parts of GRRs not previously covered by this seed were identified and then used to assemble combinations with the current seed. For each of the combinations, a coverage and a fold-change value were calculated, indicating how much the coverage increased compared to the maximum coverage obtained from individual regulatory maps, as depicted in Figure 4A. Next, from each seed combination set, only the combination with the highest fold-change was kept, while others were discarded. Therefore, each seed map provides exactly one candidate combination that could potentially improve the aetiological connections with a disease or a trait of interest. Top n (default is three) of these candidate combinations with the highest fold-change are tested for a given disease or a trait (Fig. 4D) using the method described above for individual CRE maps. Detailed description of the commands and calling parameters used here is provided in the Online user manual (https://github.com/mnikoli/GARLIC).

## Supplementary Material

Supplementary Material is available at *HMG* online.

## Supplementary Material

Supplementary Data
