# Supplementary material for: GARLIC: a bioinformatic toolkit for aetiologically connecting diseases and cell type-specific regulatory maps
Source: Hum Mol Genet. 2016 Dec 22;26(4):742–52. doi: 10.1093/hmg/ddw423 (PMC5409087; doi:10.1093/hmg/ddw423)
Supplement: Supplementary Data [file ddw423_Supp.pdf]

Supplementary Material

GARLIC: A Bioinformatic Toolkit for Etiologically Connecting Diseases and Cell Type-Specific Regulatory Maps

Miloš Nikolić[1,2], Argyris Papantonis[1], Alvaro Rada-Iglesias[1,2,*]

[1] Center for Molecular Medicine Cologne (CMMC) , Robert-Koch-Str. 21, 50931 Cologne, Germany

[2] The Cologne Cluster of Excellence in Cellular Stress Responses in Aging-associated Diseases (CECAD), Joseph-Stelzmann-Straße 26, 50931 Cologne, Germany

* Corresponding author:

Alvaro Rada-Iglesias

Junior Research Group Leader - Developmental Genomics Laboratory

Tel: +49 (0) 221 478 96 988

Email: aradaigl@uni-koeln.de

Supplementary Figures:

Fig S1. **An overview of the GARLIC DB schema.**

Fig S2. **Results for 167 diseases/traits with respect to 77 CRE maps.**

Fig S3. **Etiological connections for selected CRE maps.**

Fig S4. **Procedure to identify cell type combinations with an increased etiological contribution to complex diseases/traits.**

Fig S5. **GARLIC results using recursive splicing site (RSS) maps from HUVECs and neuronal progenitors.**

Supplementary Tables:

Table S1. **Information regarding the public DNAse-seq regulatory maps used by GARLIC.**

Table S2. **Example of GARLIC output after testing the etiological connection between 510 different diseases/traits and the CRE map from fetal heart.**

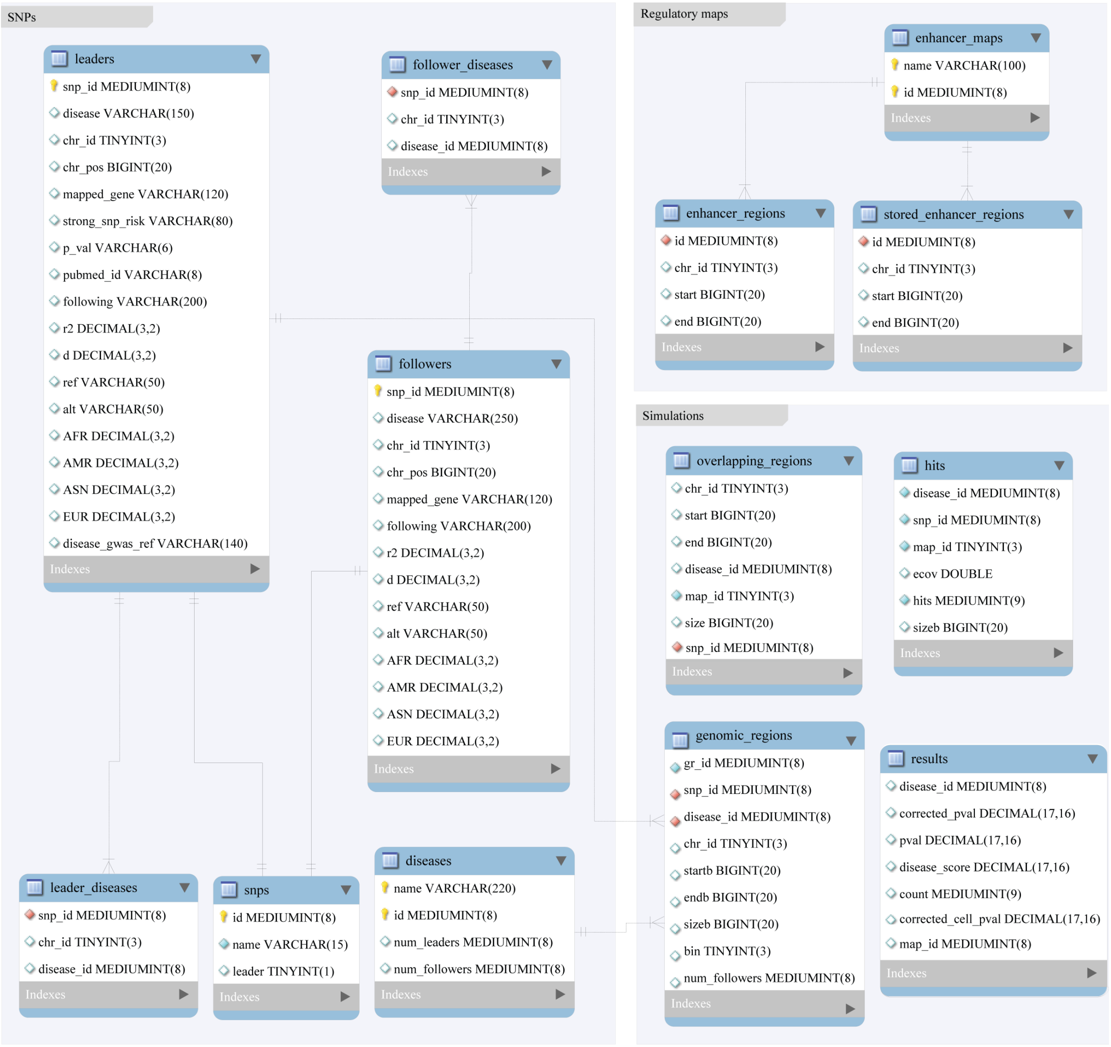

Fig S1**. An overview of the GARLIC DB schema.**

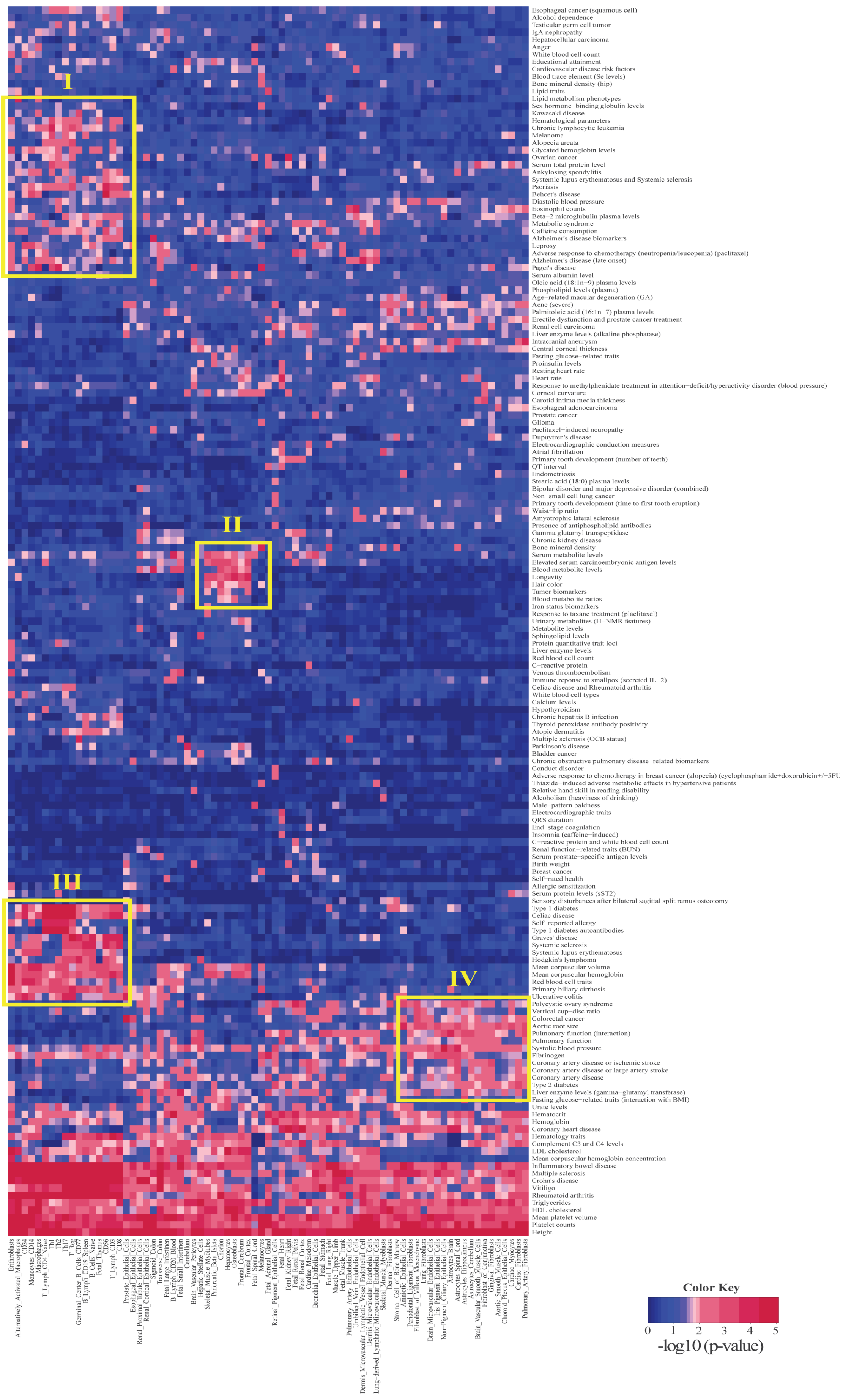
I
II
III
IV
Esophageal cancer (squamous cell)
Alcohol dependence
Testicular germ cell tumor
IgA nephropathy
Hepatocellular carcinoma
Anger
White blood cell count
Educational attainment
Cardiovascular disease risk factors
Blood trace element (Se levels)
Bone mineral density (hip)
Lipid traits
Lipid metabolism phenotypes
Sex hormone−binding globulin levels
Kawasaki disease
Hematological parameters
Chronic lymphocytic leukemia
Melanoma
Alopecia areata
Glycated hemoglobin levels
Ovarian cancer
Serum total protein level
Ankylosing spondylitis
Systemic lupus erythematosus and Systemic sclerosis
Psoriasis
Behcet's disease
Diastolic blood pressure
Eosinophil counts
Beta−2 microglubulin plasma levels
Metabolic syndrome
Caffeine consumption
Alzheimer's disease biomarkers
Leprosy
Adverse response to chemotherapy (neutropenia/leucopenia) (paclitaxel)
Alzheimer's disease (late onset)
Paget's disease
Serum albumin level
Oleic acid (18:1n−9) plasma levels
Phospholipid levels (plasma)
Age−related macular degeneration (GA)
Acne (severe)
Palmitoleic acid (16:1n−7) plasma levels
Erectile dysfunction and prostate cancer treatment
Renal cell carcinoma
Liver enzyme levels (alkaline phosphatase)
Intracranial aneurysm
Central corneal thickness
Fasting glucose−related traits
Proinsulin levels
Resting heart rate
Heart rate
Response to methylphenidate treatment in attention−deficit/hyperactivity disorder (blood pressure)
Corneal curvature
Carotid intima media thickness
Esophageal adenocarcinoma
Prostate cancer
Glioma
Paclitaxel−induced neuropathy
Dupuytren's disease
Electrocardiographic conduction measures
Atrial fibrillation
Primary tooth development (number of teeth)
QT interval
Endometriosis
Stearic acid (18:0) plasma levels
Bipolar disorder and major depressive disorder (combined)
Non−small cell lung cancer
Primary tooth development (time to first tooth eruption)
Waist−hip ratio
Amyotrophic lateral sclerosis
Presence of antiphospholipid antibodies
Gamma glutamyl transpeptidase
Chronic kidney disease
Bone mineral density
Serum metabolite levels
Elevated serum carcinoembryonic antigen levels
Blood metabolite levels
Longevity
Hair color
Tumor biomarkers
Blood metabolite ratios
Iron status biomarkers
Response to taxane treatment (placlitaxel)
Urinary metabolites (H−NMR features)
Metabolite levels
Sphingolipid levels
Protein quantitative trait loci
Liver enzyme levels
Red blood cell count
C−reactive protein
Venous thromboembolism
Immune reponse to smallpox (secreted IL−2)
Celiac disease and Rheumatoid arthritis
White blood cell types
Calcium levels
Hypothyroidism
Chronic hepatitis B infection
Thyroid peroxidase antibody positivity
Atopic dermatitis
Multiple sclerosis (OCB status)
Parkinson's disease
Bladder cancer
Chronic obstructive pulmonary disease−related biomarkers
Conduct disorder
Adverse response to chemotherapy in breast cancer (alopecia) (cyclophosphamide+doxorubicin+/−5FU
Thiazide−induced adverse metabolic effects in hypertensive patients
Relative hand skill in reading disability
Alcoholism (heaviness of drinking)
Male−pattern baldness
Electrocardiographic traits
QRS duration
End−stage coagulation
Insomnia (caffeine−induced)
C−reactive protein and white blood cell count
Renal function−related traits (BUN)
Serum prostate−specific antigen levels
Birth weight
Breast cancer
Self−rated health
Allergic sensitization
Serum protein levels (sST2)
Sensory disturbances after bilateral sagittal split ramus osteotomy
Type 1 diabetes
Celiac disease
Self−reported allergy
Type 1 diabetes autoantibodies
Graves' disease
Systemic sclerosis
Systemic lupus erythematosus
Hodgkin's lymphoma
Mean corpuscular volume
Mean corpuscular hemoglobin
Red blood cell traits
Primary biliary cirrhosis
Ulcerative colitis
Polycystic ovary syndrome
Vertical cup−disc ratio
Colorectal cancer
Aortic root size
Pulmonary function (interaction)
Pulmonary function
Systolic blood pressure
Fibrinogen
Coronary artery disease or ischemic stroke
Coronary artery disease or large artery stroke
Coronary artery disease
Type 2 diabetes
Liver enzyme levels (gamma−glutamyl transferase)
Fasting glucose−related traits (interaction with BMI)
Urate levels
Hematocrit
Hemoglobin
Coronary heart disease
Hematology traits
Complement C3 and C4 levels
LDL cholesterol
Mean corpuscular hemoglobin concentration
Inflammatory bowel disease
Multiple sclerosis
Crohn's disease
Vitiligo
Rheumatoid arthritis
Triglycerides
HDL cholesterol
Mean platelet volume
Platelet counts
Height
Color Key
0 1 2 3 4 5
-log10 (p-value)

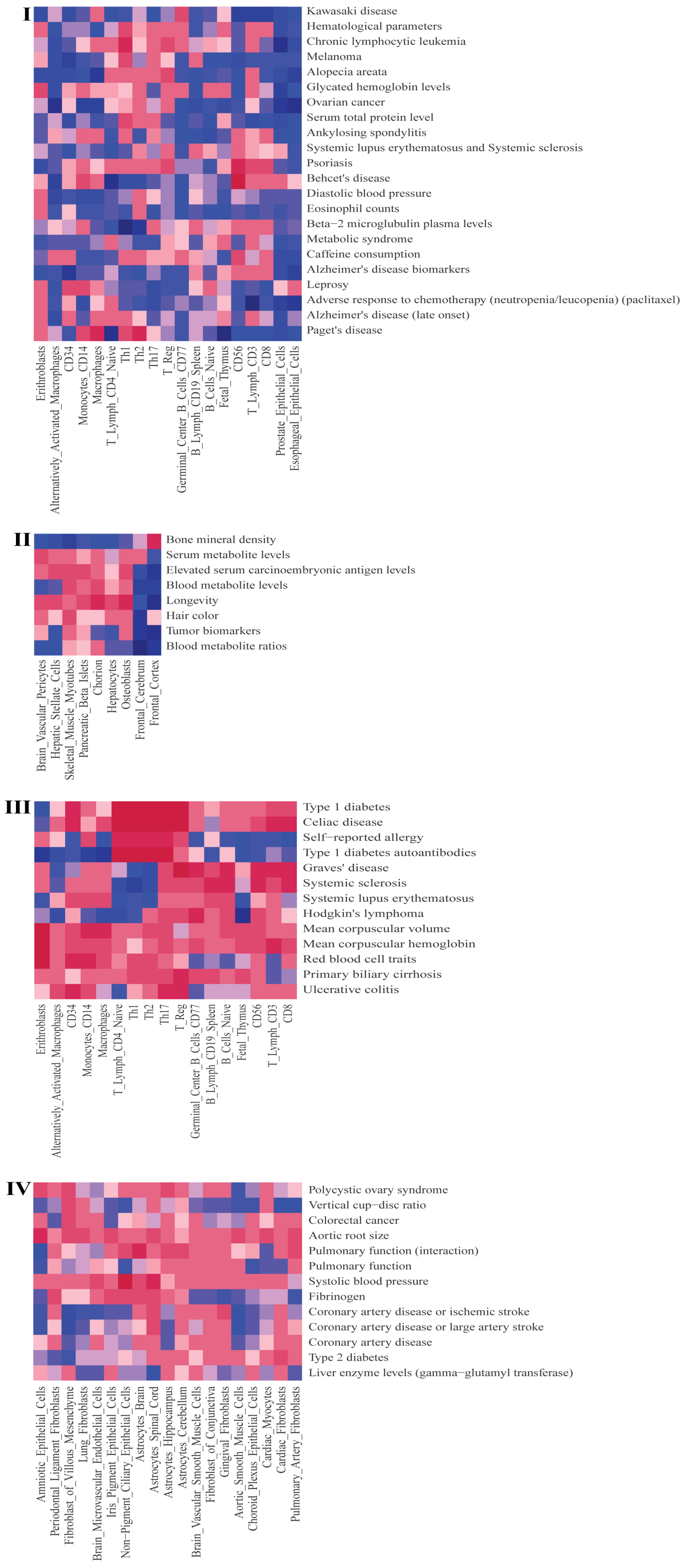
I
Kawasaki disease
Hematological parameters
Chronic lymphocytic leukemia
Melanoma
Alopecia areata
Glycated hemoglobin levels
Ovarian cancer
Serum total protein level
Ankylosing spondylitis
Systemic lupus erythematosus and Systemic sclerosis
Psoriasis
Behcet's disease
Diastolic blood pressure
Eosinophil counts
Beta−2 microglubulin plasma levels
Metabolic syndrome
Caffeine consumption
Alzheimer's disease biomarkers
Leprosy
Adverse response to chemotherapy (neutropenia/leucopenia) (paclitaxel)
Alzheimer's disease (late onset)
Paget's disease
Erithroblasts
Alternatively_Activated_Macrophages
CD34
Monocytes_CD14
Macrophages
T_Lymph_CD4_Naive
Th1
Th2
Th17
T_Reg
Germinal_Center_B_Cells_CD77
B_Lymph_CD19_Spleen
B_Cells_Naive
Fetal_Thymus
CD56
T_Lymph_CD3
CD8
Prostate_Epithelial_Cells
Esophageal_Epithelial_Cells
II
Bone mineral density
Serum metabolite levels
Elevated serum carcinoembryonic antigen levels
Blood metabolite levels
Longevity
Hair color
Tumor biomarkers
Blood metabolite ratios
Brain_Vascular_Pericytes
Hepatic_Stellate_Cells
Skeletal_Muscle_Myotubes
Pancreatic_Beta_Islets
Chorion
Hepatocytes
Osteoblasts
Frontal_Cerebrum
Frontal_Cortex
III
Type 1 diabetes
Celiac disease
Self−reported allergy
Type 1 diabetes autoantibodies
Graves' disease
Systemic sclerosis
Systemic lupus erythematosus
Hodgkin's lymphoma
Mean corpuscular volume
Mean corpuscular hemoglobin
Red blood cell traits
Primary biliary cirrhosis
Ulcerative colitis
Erithroblasts
Alternatively_Activated_Macrophages
CD34
Monocytes_CD14
Macrophages
T_Lymph_CD4_Naive
Th1
Th2
Th17
T_Reg
Germinal_Center_B_Cells_CD77
B_Lymph_CD19_Spleen
B_Cells_Naive
Fetal_Thymus
CD56
T_Lymph_CD3
CD8
IV
Polycystic ovary syndrome
Vertical cup−disc ratio
Colorectal cancer
Aortic root size
Pulmonary function (interaction)
Pulmonary function
Systolic blood pressure
Fibrinogen
Coronary artery disease or ischemic stroke
Coronary artery disease or large artery stroke
Coronary artery disease
Type 2 diabetes
Liver enzyme levels (gamma−glutamyl transferase)
Amniotic_Epithelial_Cells
Periodontal_Ligament_Fibroblasts
Fibroblast_of_Villous_Mesenchyme
Lung_Fibroblasts
Brain_Microvascular_Endothelial_Cells
Iris_Pigment_Epithelial_Cells
Non−Pigment_Ciliary_Epithelial_Cells
Astrocytes_Brain
Astrocytes_Spinal_Cord
Astrocytes_Hippocampus
Astrocytes_Cerebellum
Brain_Vascular_Smooth_Muscle_Cells
Fibroblast_of_Conjunctiva
Gingival_Fibroblasts
Aortic_Smooth_Muscle_Cells
Choroid_Plexus_Epithelial_Cells
Cardiac_Myocytes
Cardiac_Fibroblasts
Pulmonary_Artery_Fibroblasts

Fig S2. **Results for 167 diseases/traits with respect to 77 CRE maps.** The selected diseases (rows) are those displaying a significant connection (p<=0.01) with at least one of the CRE maps (columns). The statistical connection between each disease and cell type is color coded according to GARLIC *p-values*, with the most and least significant connections represented in red and blue, respectively. Four different clusters (I-IV) linking related diseases/traits and CRE maps are highlighted in yellow and showed as separate figure panels.

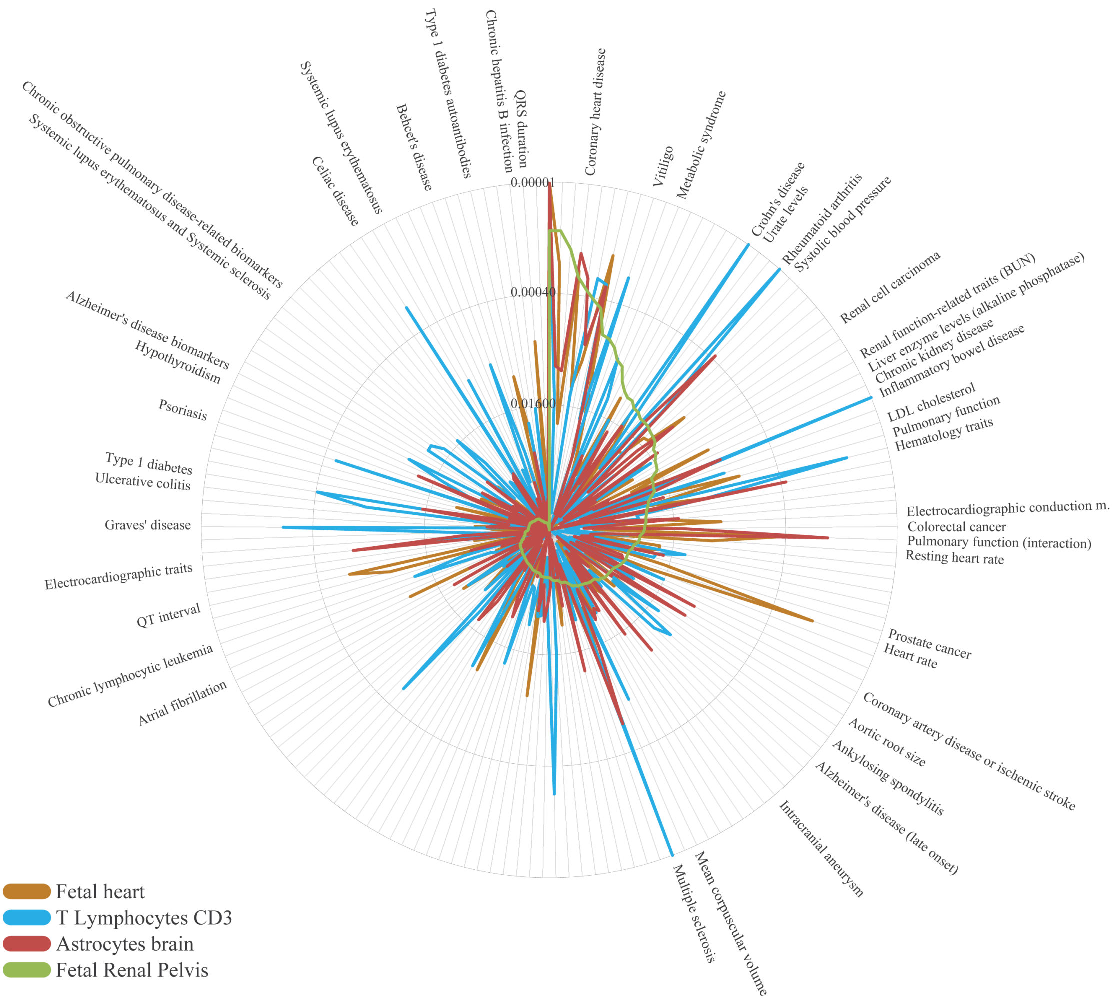

Fig S3. **Etiological connections for selected CRE maps.** Radial plot summarizing the statistical connection between four selected CRE maps (indicated in the bottom left corner) and all diseases included in the GARLIC DB for which a significant connection (p<=0.01) with at least one CRE map was found. The names of only a subset of all the investigated cell types are shown. Peaks closer to the outer border of the radial plot represent more significant connections, while those closer to the center are the least significant ones.

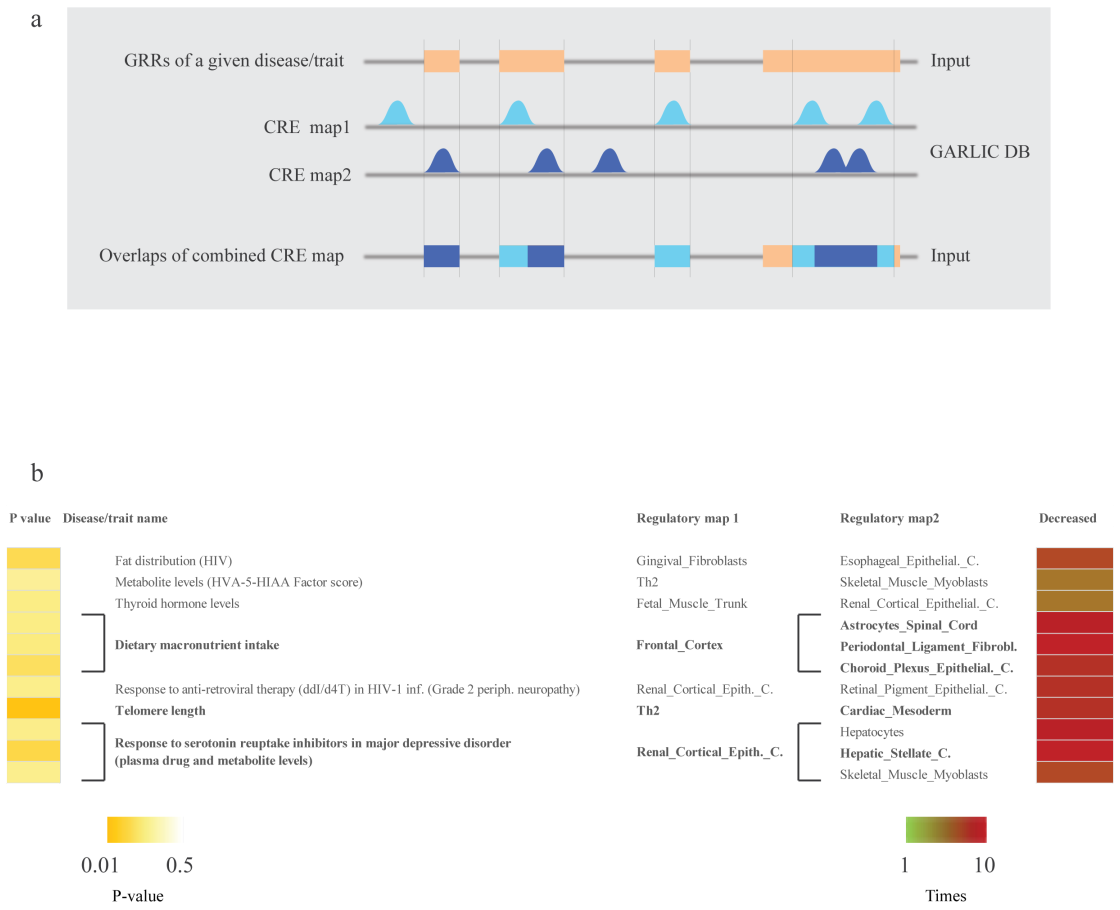

Fig S4. **Procedure to identify cell type combinations with an increased etiological contribution to complex diseases/traits. (a)** Graphical overview of how the combination of CRE maps from different cell types might increase the overall coverage of GRRs with respect to the individual CRE maps. This can then lead to a stronger etiological connection with the disease/trait under consideration. **(b)** Summary of the results obtained after testing the etiological connection of pairwise CRE map combinations with respect to 95 diseases/traits. The diseases/traits for which the *p-values* obtained with combined CRE maps were at least five times lower than the *p-values* obtained when the corresponding CRE maps were tested separately are shown. CRE map combinations that yielded *p*-values lower than those achieved when all CRE maps were tested individually are highlighted in bold. Likewise, diseases/traits for which at least one CRE map combination yielded *p-values* lower than those obtained with individual CRE maps are also highlighted in bold.

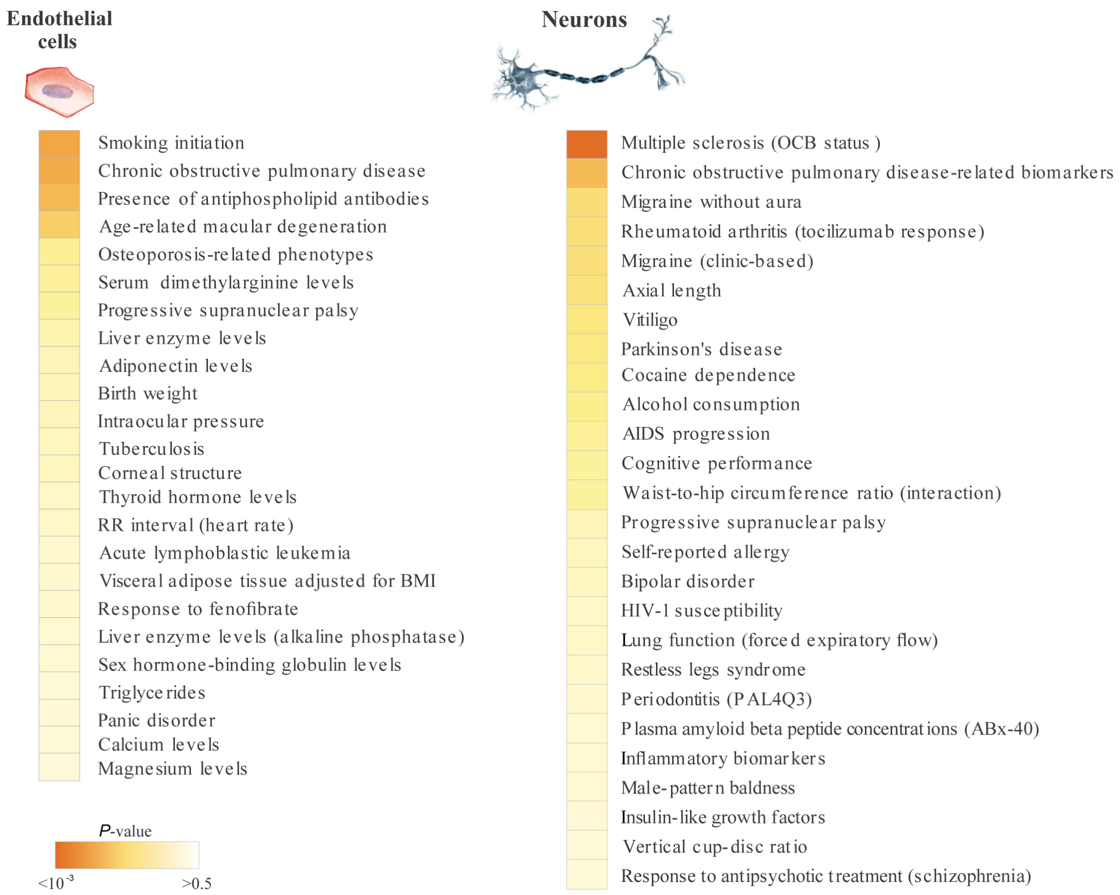

Fig S5. **GARLIC results using recursive splicing site (RSS) maps from HUVECs and neuronal progenitors.** Lists of RSSs residing in genes larger than 150 Kb were used as input for GARLIC. Heat maps illustrate the *p-values* for the listed diseases/traits associated with RSSs in each cell type.

Table S1. Information regarding the public DNAse-seq regulatory maps used by GARLIC.

Tissue/Cell Type	Experiment type	Projec	GEO_accession	Peak Calling Method *
Amniotic_Epithelial_Cells	DNAse-seq	ENCODE	GSM736631	Hotspot
Aortic_Smooth_Muscle_Cells	DNAse-seq	ENCODE	GSM816638	Hotspot
Astrocytes_Brain	DNAse-seq	ENCODE	GSM849026	Hotspot
Astrocytes_Cerebellum	DNAse-seq	ENCODE	GSM736586	Hotspot
Astrocytes_Hippocampus	DNAse-seq	ENCODE	GSM736594	Hotspot
Astrocytes_Spinal_Cord	DNAse-seq	ENCODE	GSM1014508	Hotspot
Brain_Microvascular_Endothelial_Cells	DNAse-seq	ENCODE	GSM736554	Hotspot
Brain_Vascular_Pericytes	DNAse-seq	ENCODE	GSM1024750	Hotspot
Brain_Vascular_Smooth_Muscle_Cells	DNAse-seq	ENCODE	GSM1024769	Hotspot
Bronchial_Epithelial_Cells	DNAse-seq	ENCODE	GSM1024781	Hotspot
B_Cells_Naive	DNAse-seq	ENCODE	GSM1008557	Hotspot
B_Lymph_CD19_Spleen	DNAse-seq	Roadmap	GSM701492	MACS2
B_Lymph_CD20_Blood	DNAse-seq	Roadmap	GSM701500	MACS2
Cardiac_Fibroblasts	DNAse-seq	ENCODE	GSM736568	Hotspot
Cardiac_Mesoderm	DNAse-seq	ENCODE	GSM1024759	Hotspot
Cardiac_Myocytes	DNAse-seq	ENCODE	GSM736516	Hotspot
CD34	DNAse-seq	Roadmap	GSM530657	MACS2
CD56	DNAse-seq	Roadmap	GSM665820	MACS2
CD8	DNAse-seq	Roadmap	GSM701499	MACS2
Cerebellum	DNAse-seq	ENCODE	GSM1008583	Hotspot
Chorion	DNAse-seq	ENCODE	GSM816628	Hotspot
Choroid_Plexus_Epithelial_Cells	DNAse-seq	ENCODE	GSM736597	Hotspot
Dermal_Fibroblasts	DNAse-seq	ENCODE	GSM736567	Hotspot
Dermis_Microvascular_Endothelial_Cells	DNAse-seq	ENCODE	GSM736624	Hotspot
Dermis_Microvascular_Lymphatic_Vessel_Endothelial_Cell	DNAse-seq	ENCODE	GSM736599	Hotspot
Esophageal_Epithelial_Cells	DNAse-seq	ENCODE	GSM736585	Hotspot
Fetal_Adrenal_Gland	DNAse-seq	Roadmap	GSM530653	MACS2
Fetal_Heart	DNAse-seq	Roadmap	GSM530661	MACS2
Fetal_Kidney_Right	DNAse-seq	Roadmap	GSM665810	MACS2
Fetal_Large_Intestine	DNAse-seq	Roadmap	GSM665815	MACS2
Fetal_Lung_Right	DNAse-seq	Roadmap	GSM701523	MACS2
Fetal_Muscle_Trunk	DNAse-seq	Roadmap	GSM701533	MACS2
Fetal_Renal_Cortex	DNAse-seq	Roadmap	GSM530655	MACS2
Fetal_Renal_Pelvis	DNAse-seq	Roadmap	GSM701501	MACS2
Fetal_Small_Intestine	DNAse-seq	Roadmap	GSM701530	MACS2
Fetal_Spinal_Cord	DNAse-seq	Roadmap	GSM1027339	MACS2
Fetal_Stomach	DNAse-seq	Roadmap	GSM701538	MACS2
Fetal_Thymus	DNAse-seq	Roadmap	GSM701497	MACS2
Fibroblast_of_Conjunctiva	DNAse-seq	ENCODE	GSM736547	Hotspot
Fibroblast_of_Villous_Mesenchyme	DNAse-seq	ENCODE	GSM736534	Hotspot
Frontal_Cerebrum	DNAse-seq	ENCODE	GSM1008578	Hotspot
Frontal_Cortex	DNAse-seq	ENCODE	GSM1008566	Hotspot
Germinal_Center_B_Cells_CD77	DNAse-seq	ENCODE	GSM1008579	Hotspot
Gingival_Fibroblasts	DNAse-seq	ENCODE	GSM736579	Hotspot
Hepatic_Stellate_Cells	DNAse-seq	ENCODE	GSM816672	Hotspot
Hepatocytes	DNAse-seq	ENCODE	GSM816663	Hotspot
Renal_Cortical_Epithelial_Cells	DNAse-seq	ENCODE	GSM736557	Hotspot
Iris_Pigment_Epithelial_Cells	DNAse-seq	ENCODE	GSM736615	Hotspot
Lung_Fibroblasts	DNAse-seq	ENCODE	GSM736612	Hotspot
Lung-derived_Microvascular_Endothelial_Cells	DNAse-seq	ENCODE	GSM736627	Hotspot
Foreskin_Melanocytes	DNAse-seq	ENCODE	GSM1027312	MACS2
Monocytes_CD14	DNAse-seq	Roadmap	GSM665840	MACS2
Fetal_Skeletal_Muscle_Upper_Limb	DNAse-seq	Roadmap	GSM701522	Hotspot
Non-Pigment_Ciliary_Epithelial_Cells	DNAse-seq	ENCODE	GSM736621	Hotspot
Osteoblasts	DNAse-seq	ENCODE	GSM816654	Hotspot
Pancreatic_Beta_Islets	DNAse-seq	ENCODE	GSM816660	Hotspot
Periodontal_Ligament_Fibroblasts	DNAse-seq	ENCODE	GSM736632	Hotspot
Prostate_Epithelial_Cells	DNAse-seq	ENCODE	GSM1024743	Hotspot
Pulmonary_Artery_Endothelial_Cells	DNAse-seq	ENCODE	GSM1024763	Hotspot
Pulmonary_Artery_Fibroblasts	DNAse-seq	ENCODE	GSM736614	Hotspot

Renal_Proximal_Tubule_Epithelial_Cells	DNAse-seq	ENCODE	GSM736543	Hotspot
Retinal_Pigment_Epithelial_Cells	DNAse-seq	ENCODE	GSM736630	Hotspot
Skeletal_Muscle_Myoblasts	DNAse-seq	ENCODE	GSM736560	Hotspot
Skeletal_Muscle_Myotubes	DNAse-seq	ENCODE	GSM816651	Hotspot
Stromal_Cell_of_Bone_Marrow	DNAse-seq	ENCODE	GSM1024757	Hotspot
Th1	DNAse-seq	ENCODE	GSM1024749	Hotspot
Th17	DNAse-seq	ENCODE	GSM1024790	Hotspot
Th2	DNAse-seq	Roadmap	GSM1014522	MACS2
T_Lymph_CD3	DNAse-seq	Roadmap	GSM665837	Hotspot
T_Lymph_CD4_Naive	DNAse-seq	Roadmap	GSM1014537	Hotspot
T_Reg	DNAse-seq	Roadmap	GSM1024744	Hotspot
Umbilical_Vein_Endothelial_Cells	DNAse-seq	ENCODE	GSM736575	Hotspot
Transverse_Colon	DNAse-seq	ENCODE	ENCFF693FUF	Hotspot
Sigmoid_Colon	DNAse-seq	ENCODE	ENCFF693FUF	Hotspot
Macrophages	DNAse-seq	EGA-Blueprint	ERX616976	Hotspot
Alternatively_Activated_Macrophages	DNAse-seq	EGA-Blueprint	ERX947561	Hotspot
Erythroblasts	DNAse-seq	EGA-Blueprint	ERX1488565	Hotspot

* DNAse-seq peaks were identified using MACS2 or Hotspot. For MACS2, afalse-discovery rate (FDR) cutoff of 0.01 was applied. For Hotspot, different cutoffs were used by different consortia (0.01 for ENCODE and Roadmap; 0.05 for BLUEPRINT).

Table S2. Example of GARLIC output after testing the etiological connection between 510 different diseases/ traits and the CRE map from fetal heart.

Disease_ID	Disease_Name	Corrected_P_Value	P_Value	Disease_Score	Num_Leaders	Num_Followers	Count
391	Height	0.005099949	0.0000099999000000	0.0005056190000000	374	8597	0
235	Mean platelet volume	0.01699983	0.0000899991000000	0.0065379098000000	45	956	8
385	Heart rate	0.01699983	0.0000999990000000	0.0074669603000000	37	854	9
954	Hematocrit	0.018359816	0.0001499985000000	0.0639992957000000	8	34	14
155	Coronary heart disease	0.018359816	0.0001799982000000	0.0018513565000000	119	2628	17
493	Pulmonary function (interaction)	0.037399626	0.0004399956000000	0.0064617445000000	42	1148	43
941	Electrocardiographic traits	0.083784876	0.0011499885000000	0.0176667705000000	18	399	114
283	Pulmonary function	0.093074069	0.0014599854000000	0.0040957747000000	58	1722	145
412	QRS duration	0.109365573	0.0019299807000000	0.0167426505000000	21	277	192
723	Liver enzyme levels (alkaline phosphatase)	0.133570093	0.0027799722000000	0.0255892986000000	14	446	277
164	Coronary artery disease	0.133570093	0.0030199698000000	0.0116936977000000	23	746	301
435	Electrocardiographic conduction measures	0.133570093	0.0033999660000000	0.0258095636000000	12	371	339
236	Platelet counts	0.133570093	0.0034299657000000	0.0029049503000000	75	1557	342
838	Response to methylphenidate treatment in attention-deficit/hyperactivity disorder (blood pressure)	0.133570093	0.0039399606000000	0.0579510827000000	7	123	393
413	QT interval	0.133570093	0.0042599574000000	0.0032262099000000	70	1119	425
953	Hemoglobin	0.133570093	0.0043399566000000	0.0473055885000000	8	33	433
89	Resting heart rate	0.133570093	0.0045699543000000	0.0359226242000000	10	241	456
448	Multiple sclerosis	0.133570093	0.0048699513000000	0.0012135853000000	153	2504	486
742	Proinsulin levels	0.133570093	0.0054599454000000	0.0351759940000000	10	176	545
34	HDL cholesterol	0.133570093	0.0054699453000000	0.0017298207000000	111	2380	546
589	Insomnia (caffeine-induced)	0.133570093	0.0054999450000000	0.0463069981000000	9	72	549
641	Atrial fibrillation	0.141407677	0.0060999390000000	0.0174424519000000	17	302	609
546	Complement C3 and C4 levels	0.15588105	0.0070299297000000	0.0374336953000000	10	153	702
35	Triglycerides	0.177223228	0.0083399166000000	0.0025291561000000	73	1471	833
527	Inflammatory bowel disease	0.195563429	0.0098899011000000	0.0016797058000000	116	3299	988
296	Crohn's disease	0.195563429	0.0099699003000000	0.0011677097000000	149	4113	996
124	Type 2 diabetes	0.229119931	0.0121298787000000	0.0011426432000000	157	3387	1212
339	Primary tooth development (number of teeth)	0.233322667	0.0128098719000000	0.0166178926000000	16	310	1280
514	Renal cell carcinoma	0.292455696	0.0166298337000000	0.0398194590000000	8	197	1662
72	Prostate cancer	0.326566734	0.0192098079000000	0.0019704111000000	95	1913	1920
552	Vitiligo	0.329687026	0.0200397996000000	0.0055709001000000	37	718	2003
869	Erectile dysfunction and prostate cancer treatment	0.353330842	0.0221697783000000	0.0112007969000000	23	344	2216
623	Fasting glucose-related traits (interaction with BMI)	0.374481969	0.0249797502000000	0.0075554229000000	31	415	2497
139	Anger	0.374481969	0.0253297467000000	0.0173390997000000	14	314	2532
214	LDL cholesterol	0.374481969	0.0256997430000000	0.0022219635000000	85	1694	2569
490	Urate levels	0.381842335	0.0279097209000000	0.0036665868000000	55	1104	2790
724	Liver enzyme levels (gamma-glutamyl transferase)	0.381842335	0.0283497165000000	0.0093273588000000	26	421	2834
461	Palmitoleic acid (16:1n-7) plasma levels	0.381842335	0.0285797142000000	0.0182019791000000	15	306	2857
563	Polycystic ovary syndrome	0.381842335	0.0291997080000000	0.0172190593000000	13	191	2919
732	Systolic blood pressure	0.384486399	0.0307496925000000	0.0078503748000000	28	671	3074
62	Rheumatoid arthritis	0.384486399	0.0309096909000000	0.0011185757000000	156	2675	3090
345	Testicular germ cell tumor	0.393181782	0.0323796762000000	0.0111732012000000	21	547	3237
478	Hematology traits	0.398863453	0.0336296637000000	0.0114780614000000	19	195	3362
701	Ankylosing spondylitis	0.42136912	0.0366196338000000	0.0092529712000000	24	532	3661
43	PR segment	0.42136912	0.0371796282000000	0.0302136267000000	8	282	3717
266	Waist-hip ratio	0.435739643	0.0395796042000000	0.0178912042000000	13	339	3957
177	Bone mineral density	0.435739643	0.0403195968000000	0.0017638361000000	101	3477	4031
141	Lipid traits	0.435739643	0.0416595834000000	0.0103266206000000	23	511	4165
487	End-stage coagulation	0.435739643	0.0426395736000000	0.0133449428000000	19	187	4263
425	Multiple myeloma (IgH translocation)	0.435739643	0.0427195728000000	0.0096958099000000	23	382	4271
766	Esophageal cancer	0.441695583	0.0441695583000000	0.0205161471000000	13	171	4416
747	Type 1 diabetes autoantibodies	0.45850503	0.0467495325000000	0.0096030382000000	22	419	4674
146	Chronic kidney disease	0.482859322	0.0501794982000000	0.0061966167000000	31	765	5017
824	Insulin-like growth factors	0.487924431	0.0533094669000000	0.0474778552000000	7	76	5330
98	Glycated hemoglobin levels	0.487924431	0.0537694623000000	0.0090380617000000	26	321	5376
685	Lipid metabolism phenotypes	0.487924431	0.0551494485000000	0.0059715649000000	34	875	5514
267	Alcohol consumption	0.487924431	0.0554694453000000	0.0109868223000000	21	293	5546
332	Blood trace element (Se levels)	0.487924431	0.0554894451000000	0.0361655390000000	10	175	5548
731	Diastolic blood pressure	0.488160743	0.0568394316000000	0.0073321586000000	28	710	5683
446	Beta-2 microglubulin plasma levels	0.488160743	0.0584894151000000	0.0465088902000000	7	70	5848
369	Adverse response to chemotherapy (neutropenia/leucopenia) (paclitaxel)	0.488160743	0.0603793962000000	0.0569769897000000	8	73	6037
166	Coronary artery disease or large artery stroke	0.488160743	0.0603893961000000	0.0142634006000000	17	514	6038
774	Dialysis-related mortality	0.488160743	0.0605593944000000	0.0089751889000000	25	638	6055
550	Intracranial aneurysm	0.488160743	0.0612593874000000	0.0187071910000000	12	519	6125
471	Metabolite levels (HVA-5-HIAA Factor score)	0.492576613	0.0627793722000000	0.0345613532000000	12	38	6277
496	Mean corpuscular volume	0.499795002	0.0646793532000000	0.0060999859000000	33	685	6467
793	Caffeine consumption	0.500708426	0.0657793422000000	0.0251872503000000	10	119	6577
665	Metabolic syndrome	0.53204468	0.0709392906000000	0.0053238293000000	38	537	7093
935	Fasting glucose-related traits	0.593430066	0.0808191918000000	0.0128656707000000	17	242	8081
538	Serum total protein level	0.593430066	0.0822091779000000	0.0365849700000000	7	99	8220
657	Hypothyroidism	0.593430066	0.0847491525000000	0.0099046733000000	19	479	8474
957	Bone mineral density (hip)	0.593430066	0.0855591444000000	0.0091073869000000	22	448	8555
421	Acute lymphoblastic leukemia (childhood)	0.593430066	0.0861791382000000	0.0059747835000000	32	544	8617
545	Primary biliary cirrhosis	0.593430066	0.0862891371000000	0.0068496192000000	30	658	8628
378	Amyotrophic lateral sclerosis	0.593430066	0.0872691273000000	0.0036129757000000	52	705	8726
462	Stearic acid (18:0) plasma levels	0.629910806	0.0938690613000000	0.0205101148000000	11	142	9386
531	Bipolar disorder (mood-incongruent)	0.684699251	0.1054289457000000	0.0124773206000000	16	534	10542
727	Cardiovascular disease risk factors	0.684699251	0.1058389416000000	0.0091171860000000	25	229	10583
534	Stroke (ischemic)	0.684699251	0.1074189258000000	0.0295151274000000	8	101	10741
279	Metabolite levels (Pyroglutamine)	0.684699251	0.1075289247000000	0.0082499770000000	23	504	10752
521	Chronic obstructive pulmonary disease-related biomarkers	0.684699251	0.1089389106000000	0.0056827923000000	34	472	10893
813	Celiac disease and Rheumatoid arthritis	0.684699251	0.1100888991000000	0.0136990095000000	15	498	11008
684	Type 1 diabetes	0.690821353	0.1128588714000000	0.0024273850000000	72	1135	11285
865	Cerebrospinal AB1-42 levels	0.690821353	0.1146488535000000	0.0352457372000000	7	55	11464
565	Response to fenofibrate	0.690821353	0.1154588454000000	0.0316613054000000	7	24	11545
303	Self-reported allergy	0.690821353	0.1166288337000000	0.0053286145000000	35	550	11662
918	Bipolar disorder and major depressive disorder (combined)	0.690821353	0.1180588194000000	0.0208154827000000	9	211	11805
777	Neutrophil count	0.690821353	0.1211587884000000	0.0085438833000000	22	418	12115
143	Cognitive decline (age-related)	0.690821353	0.1220187798000000	0.0241663866000000	11	92	12201
206	Adiponectin levels	0.690821353	0.1222987770000000	0.0044672911000000	45	920	12229
642	Wilms tumor	0.690821353	0.1236987630000000	0.0298324456000000	7	106	12369
360	Adverse response to chemotherapy (neutropenia/leucopenia) (carboplatin)	0.690821353	0.1246187538000000	0.0262145174000000	9	51	12461
733	Gamma glutamyl transpeptidase	0.706973575	0.1289187108000000	0.0386030807000000	7	61	12891
495	Mean corpuscular hemoglobin concentration	0.721751293	0.1330286697000000	0.0183973662000000	12	214	13302
597	Age-related macular degeneration (GA)	0.73598639	0.1372886271000000	0.0222028439000000	9	116	13728
523	Non-small cell lung cancer	0.73598639	0.1385386146000000	0.0138409011000000	16	176	13853
218	Liver enzyme levels (alanine transaminase)	0.736172827	0.1404585954000000	0.0162917961000000	15	194	14045
984	Eosinophil counts	0.736172827	0.1422585774000000	0.0303718325000000	7	79	14225
208	Digestive system disease (Barrett's esophagus and esophageal adenocarcinoma combined)	0.736172827	0.1452585474000000	0.0210489533000000	10	286	14525
306	Migraine with aura	0.736172827	0.1452985470000000	0.0081440634000000	23	302	14529
715	IgE grass sensitization	0.736172827	0.1458485415000000	0.0192335018000000	9	173	14584
891	Alopecia areata	0.736172827	0.1502984970000000	0.0235127544000000	8	167	15029
677	Sphingolipid levels	0.736172827	0.1521584784000000	0.0222742540000000	11	78	15215
537	Serum albumin level	0.736172827	0.1522484775000000	0.0227992218000000	9	101	15224
165	Coronary artery disease or ischemic stroke	0.736172827	0.1528284717000000	0.0151255480000000	15	395	15282
847	Response to antipsychotic treatment in schizophrenia (working memory)	0.736172827	0.1530084699000000	0.0184450121000000	11	495	15300
377	Graves' disease	0.737729071	0.1547784522000000	0.0079679853000000	23	385	15477
305	Migraine - clinic-based	0.741366945	0.1618583814000000	0.0046696582000000	37	617	16185
48	Plasma omega-6 polyunsaturated fatty acid levels (arachidonic acid)	0.741366945	0.1624083759000000	0.0290965747000000	7	244	16240
160	Alcohol dependence	0.741366945	0.1628983710000000	0.0057836600000000	29	400	16289
288	Red blood cell traits	0.741366945	0.1629783702000000	0.0028122433000000	60	965	16297
403	Body mass index (non-asthmatics)	0.741366945	0.1636783632000000	0.0202188263000000	9	192	16367
340	Primary tooth development (time to first tooth eruption)	0.741366945	0.1654983450000000	0.0133285109000000	15	159	16549
291	HIV-1 susceptibility	0.741366945	0.1675983240000000	0.0146092572000000	16	233	16759
900	Vertical cup-disc ratio	0.741366945	0.1690583094000000	0.0194322314000000	9	174	16905
588	Osteoarthritis	0.741366945	0.1700382996000000	0.0225443886000000	8	594	17003
122	Intraocular pressure	0.741366945	0.1700782992000000	0.0145133581000000	13	132	17007
459	Oleic acid (18:1n-9) plasma levels	0.742517998	0.1717982820000000	0.0204876973000000	9	39	17179
494	Mean corpuscular hemoglobin	0.745060049	0.1751082489000000	0.0077029339000000	25	510	17510
864	Psoriasis	0.745060049	0.1753082469000000	0.0056110722000000	31	608	17530
197	Alzheimer's disease (late onset)	0.745519817	0.1768782312000000	0.0026577515000000	61	1015	17687
209	Esophageal adenocarcinoma	0.750110532	0.1794382056000000	0.0221796703000000	8	178	17943
428	Central corneal thickness	0.763292367	0.1840881591000000	0.0208842516000000	9	120	18408
714	Allergic rhinitis	0.765814922	0.1861981380000000	0.0196921356000000	9	93	18619
246	Lung function (forced expiratory flow between 25% and 75% of forced vital capacity)	0.769317107	0.1885581144000000	0.0172235936000000	12	248	18855
640	Electroencephalographic traits in alcoholism	0.781789801	0.1931480685000000	0.0234656024000000	8	93	19314
694	Prion diseases	0.800811523	0.1997180028000000	0.0237448664000000	11	132	19971
573	Sex hormone-binding globulin levels	0.800811523	0.2009879901000000	0.0073923011000000	24	283	20098
202	Serum dimethylarginine levels (symmetric)	0.806266356	0.2039379606000000	0.0061155399000000	30	130	20393
394	Alzheimer's disease biomarkers	0.817359218	0.2087579124000000	0.0106030355000000	20	197	20875

767	Paget's disease	0.817359218	0.2115478845000000	0.0208292272000000	11	80	21154
718	Leprosy	0.817359218	0.2130178698000000	0.0204039359000000	9	202	21301
579	C-reactive protein and white blood cell count	0.817359218	0.2141278587000000	0.0181548322000000	9	190	21412
441	Pubertal anthropometrics	0.817359218	0.2158278417000000	0.0066255254000000	24	620	21582
882	Magnesium levels	0.817359218	0.2191878081000000	0.0191125812000000	9	60	21918
707	Chronic obstructive pulmonary disease	0.817359218	0.2198378016000000	0.0108879209000000	16	156	21983
624	Fasting insulin-related traits (interaction with BMI)	0.817359218	0.2204677953000000	0.0089276891000000	20	265	22046
259	Fibrinogen	0.817359218	0.2211677883000000	0.0050794542000000	31	830	22116
22	Elevated serum carcinoembryonic antigen levels	0.822632781	0.2242077579000000	0.0166651702000000	11	61	22420
201	Serum dimethylarginine levels (asymmetric/symetric ratio)	0.830007784	0.2290577094000000	0.0063049620000000	24	43	22905
45	Colorectal cancer	0.830007784	0.2314676853000000	0.0024508892000000	64	1600	23146
314	Sexual dimorphism in anthropometric traits	0.830007784	0.2327176728000000	0.0229411117000000	10	95	23271
169	Antibody status in Tripanosoma cruzi seropositivity	0.830007784	0.2327276727000000	0.0085253990000000	20	456	23272
716	White blood cell count	0.84250482	0.2381176188000000	0.0115636978000000	16	276	23811
676	Phospholipid levels (plasma)	0.84250482	0.2405775942000000	0.0029990351000000	49	552	24057
116	Urinary metabolites (H-NMR features)	0.84250482	0.2433475665000000	0.0100371273000000	17	448	24334
962	Folate pathway vitamin levels	0.84250482	0.2441675583000000	0.0193667023000000	11	131	24416
823	Aortic root size	0.84250482	0.2463775362000000	0.0155152460000000	12	372	24637
914	Optic nerve measurement (disc area)	0.84250482	0.2471875281000000	0.0249317144000000	7	145	24718
520	Response to fenofibrate (adiponectin levels)	0.84250482	0.2480175198000000	0.0145870913000000	12	116	24801
473	Metabolite levels (HVA/MHPG ratio)	0.84250482	0.2494475055000000	0.0066004602000000	25	216	24944
430	Refractive error	0.859602942	0.2577974220000000	0.0061758052000000	27	732	25779
730	Carotid intima media thickness	0.859602942	0.2592874071000000	0.0224992820000000	8	155	25928
484	Corneal structure	0.859602942	0.2600573994000000	0.0055261887000000	31	571	26005
1016	Black vs. red hair color	0.859602942	0.2623273767000000	0.0266428060000000	7	17	26232
906	Nasopharyngeal carcinoma	0.859602942	0.2629373706000000	0.0196179781000000	11	235	26293
617	Immune reponse to smallpox (secreted IL-2)	0.870402124	0.2679473205000000	0.0097805739000000	17	415	26794
470	Metabolite levels (HVA)	0.871402942	0.2708472915000000	0.0147195776000000	13	14	27084
216	Psychosis (atypical)	0.871402942	0.2732072679000000	0.0216716537000000	10	323	27320
84	Cervical cancer	0.871402942	0.2750172498000000	0.0109671522000000	16	201	27501
695	Anticoagulant levels	0.871402942	0.2776872231000000	0.0192664061000000	8	250	27768
576	Renal function-related traits (sCR)	0.871402942	0.2783072169000000	0.0185067658000000	9	60	27830
399	Attention deficit hyperactivity disorder (combined symptoms)	0.871402942	0.2785072149000000	0.0230174615000000	10	66	27850
302	Allergic sensitization	0.881233871	0.2833771662000000	0.0150466858000000	12	152	28337
400	Attention deficit hyperactivity disorder (hyperactivity-impulsivity symptoms)	0.881631184	0.2857971420000000	0.0198488275000000	10	180	28579
330	Educational attainment	0.881631184	0.2904670953000000	0.0114660705000000	13	436	29046
485	Depression (quantitative trait)	0.881631184	0.2907370926000000	0.0127336616000000	13	84	29073
268	Behavioural disinhibition (generation interaction)	0.881631184	0.2919770802000000	0.0077220077000000	20	225	29197
320	DNA methylation (parent-of-origin)	0.881631184	0.2927070729000000	0.0244617225000000	7	192	29270
754	Systemic sclerosis	0.881631184	0.2938770612000000	0.0093868543000000	18	284	29387
4	Celiac disease	0.882291177	0.2958270417000000	0.0022259551000000	64	1033	29582
548	Esophageal cancer (squamous cell)	0.884159182	0.2981870181000000	0.0240817710000000	7	187	29818
562	Tourette syndrome	0.884480829	0.3028669713000000	0.0224908119000000	7	33	30286
427	Hypertriglyceridemia	0.884480829	0.3048469515000000	0.0129390796000000	12	195	30484
200	Serum dimethylarginine levels (asymmetric)	0.884480829	0.3051669483000000	0.0073532808000000	21	66	30516
152	Systolic blood pressure (alcohol consumption interaction)	0.884480829	0.3097369026000000	0.0167457196000000	9	156	30973
743	Insulin resistance/response	0.884480829	0.3114668853000000	0.0125584786000000	12	247	31146
372	Age-related macular degeneration	0.884480829	0.3130568694000000	0.0027361979000000	55	689	31305
799	Iron status biomarkers	0.884480829	0.3131068689000000	0.0052967878000000	30	290	31310
298	Sensory disturbances after bilateral sagittal split ramus osteotomy	0.884480829	0.3142868571000000	0.0222870337000000	7	6	31428
607	Testosterone levels	0.884480829	0.3168368316000000	0.0237471019000000	7	50	31683
581	Hepatocellular carcinoma	0.884480829	0.3170868291000000	0.0228135963000000	8	223	31708
205	Breast cancer	0.884480829	0.3190768092000000	0.0013411033000000	110	2466	31907
227	Tuberculosis	0.884480829	0.3191068089000000	0.0036818036000000	39	604	31910
13	Corneal curvature	0.884937637	0.3210067899000000	0.0108045745000000	16	453	32100
1004	Liver enzyme levels	0.886360285	0.3239367606000000	0.0173966719000000	11	319	32393
264	Multiple myeloma	0.886360285	0.3252367476000000	0.0179633670000000	9	258	32523
492	Dental caries	0.886360285	0.3267367326000000	0.0022827476000000	62	1003	32673
778	AIDS progression	0.887930803	0.3290567094000000	0.0166228124000000	9	158	32905
16	Birth weight	0.897268922	0.3342766572000000	0.0156987915000000	11	98	33427
660	Kawasaki disease	0.908155204	0.3425365746000000	0.0080939252000000	19	421	34253
132	Response to cytidine analogues (gemcitabine)	0.908155204	0.3426165738000000	0.0182835587000000	10	87	34261
1034	Tonometry	0.908155204	0.3477665223000000	0.0116179670000000	13	368	34776
525	Lipoprotein-associated phospholipase A2 activity and mass	0.908155204	0.3486665133000000	0.0100373459000000	14	194	34866
670	Hypertension	0.908155204	0.3488665113000000	0.0070503219000000	23	387	34886
985	Myocardial infarction (early onset)	0.908155204	0.3490165098000000	0.0166010548000000	9	424	34901
88	Post-traumatic stress disorder	0.910483281	0.3516964830000000	0.0201092749000000	8	37	35169
17	Blood pressure	0.942923904	0.3660763392000000	0.0016487316000000	84	2664	36607
679	Response to statin therapy (LDL-C)	0.945822702	0.3690563094000000	0.0204969906000000	8	166	36905
114	Response to anti-retroviral therapy (ddI/d4T) in HIV-1 infection (Grade 2 peripheral neuropathy)	0.946242518	0.3724362756000000	0.0116274755000000	13	134	37243
760	Longevity	0.946242518	0.3741262587000000	0.0034525635000000	43	841	37412
37	Lung cancer	0.946242518	0.3747862521000000	0.0066926334000000	22	637	37478
816	F-cell distribution	0.954645133	0.3799862001000000	0.0132852765000000	11	296	37998
637	Response to angiotensin II receptor blocker therapy	0.955663085	0.3858661413000000	0.0084422675000000	20	373	38586
486	Uric acid levels	0.955663085	0.3859761402000000	0.0044680739000000	33	640	38597
119	Plasma amyloid beta peptide concentrations (ABx-42)	0.955663085	0.3874561254000000	0.0204191643000000	8	63	38745
100	Serum metabolite levels	0.955663085	0.3894361056000000	0.0093278960000000	14	498	38943
350	Menarche (age at onset)	0.955663085	0.3927960720000000	0.0022482433000000	63	1776	39279
912	Optic nerve measurement (cup area)	0.955663085	0.3932660673000000	0.0177863596000000	8	100	39326
15	Age-related nuclear cataracts	0.955663085	0.3951460485000000	0.0094839548000000	15	175	39514
633	Economic and political preferences (feminism/equality)	0.955663085	0.3960160398000000	0.0152760351000000	10	150	39601
7	Melanoma	0.955663085	0.3972560274000000	0.0070974661000000	21	425	39725
269	Illicit drug use	0.958934073	0.4004959950000000	0.0120714540000000	10	64	40049
543	Type 1 diabetes nephropathy	0.9622376	0.4047359526000000	0.0173659690000000	10	115	40473
663	Pulmonary function decline	0.9622376	0.4066059339000000	0.0030946398000000	39	333	40660
154	Fractional exhaled nitric oxide (childhood)	0.9622376	0.4075359246000000	0.0101651758000000	16	161	40753
46	Ulcerative colitis	0.966616601	0.4112858871000000	0.0013975494000000	102	2318	41128
880	Non-alcoholic fatty liver disease histology (lobular)	0.972663209	0.4157658423000000	0.0114882728000000	13	120	41576
564	Glioma	0.973321774	0.4179558204000000	0.0160964045000000	10	135	41795
130	Breast cancer (early onset)	0.979122209	0.4255157448000000	0.0100246338000000	14	214	42551
308	Paclitaxel-induced neuropathy	0.979122209	0.4278657213000000	0.0180108659000000	10	114	42786
956	Hematological parameters	0.979122209	0.4288657113000000	0.0133425890000000	11	105	42886
150	Mean arterial pressure (alcohol consumption interaction)	0.979122209	0.4296957030000000	0.0137472156000000	9	17	42969
615	Immune reponse to smallpox (secreted IL-12p40)	0.979122209	0.4306156938000000	0.0166153488000000	10	456	43061
429	Response to irinotecan and platinum-based chemotherapy in non-small-cell lung cancer	0.979122209	0.4319656803000000	0.0223366090000000	7	134	43196
199	Bladder cancer	0.988432474	0.4387856121000000	0.0082155893000000	18	421	43878
578	Lung adenocarcinoma	0.988432474	0.4433255667000000	0.0140672261000000	9	550	44332
300	Homocysteine levels	0.988432474	0.4434155658000000	0.0064659348000000	19	309	44341
755	Restless legs syndrome	0.988432474	0.4438255617000000	0.0182288827000000	8	195	44382
639	Response to TNF-alpha inhibitors in rheumatoid arthritis	1	0.4561854381000000	0.0200680371000000	7	71	45618
272	Periodontitis (CDC/AAP)	1	0.4645653543000000	0.0050036861000000	26	150	46456
278	Metabolite levels (Dihydroxy docosatrienoic acid)	1	0.4648053519000000	0.0069832916000000	20	271	46480
450	Thyroid hormone levels	1	0.4663853361000000	0.0056456598000000	26	616	46638
765	Response to antineoplastic agents	1	0.4777952220000000	0.0142533280000000	9	251	47779
295	Major depressive disorder	1	0.4778652213000000	0.0016220017000000	77	858	47786
931	Hematological and biochemical traits	1	0.4781952180000000	0.0056013734000000	24	327	47819
434	Brain connectivity	1	0.4801751982000000	0.0141828320000000	9	192	48017
555	Neuroblastoma	1	0.4805851941000000	0.0185667177000000	7	119	48058
418	Estradiol plasma levels (breast cancer)	1	0.4848551514000000	0.0069010206000000	18	264	48485
417	Alzheimer's disease (cognitive decline)	1	0.4852351476000000	0.0024547587000000	44	140	48523
117	Preschool internalizing problems	1	0.4880951190000000	0.0102813807000000	13	193	48809
509	White matter integrity	1	0.4891651083000000	0.0132011628000000	9	191	48916
392	Obesity	1	0.4932150678000000	0.0022068042000000	61	1143	49321
230	Menopause (age at onset)	1	0.4947150528000000	0.0038583326000000	35	703	49471
162	Schizophrenia or bipolar disorder	1	0.4998550014000000	0.0184455219000000	7	157	49985
442	Presence of antiphospholipid antibodies	1	0.5019849802000000	0.0108061529000000	13	225	50198
472	Metabolite levels (HVA/5-HIAA ratio)	1	0.5020849792000000	0.0134483029000000	9	84	50208
212	Disc degeneration (lumbar)	1	0.5059449406000000	0.0064288113000000	20	423	50594
995	Attention deficit hyperactivity disorder (time to onset)	1	0.5066749333000000	0.0093693365000000	11	64	50667
233	Relative hand skill in reading disability	1	0.5104948951000000	0.0146892126000000	10	134	51049
140	Asthma and hay fever	1	0.5115048850000000	0.0055889512000000	21	340	51150
541	Response to taxane treatment (placlitaxel)	1	0.5128248718000000	0.0095386921000000	12	114	51282
115	Response to anti-retroviral therapy (ddI/d4T) in HIV-1 infection (Grade 3 peripheral neuropathy)	1	0.5151548485000000	0.0080553011000000	14	197	51515
351	Venous thromboembolism	1	0.5217147829000000	0.0100158481000000	12	67	52171
397	Preeclampsia	1	0.5266347337000000	0.0065522462000000	18	451	52663
620	Hippocampal atrophy	1	0.5326946731000000	0.0044311794000000	31	650	53269
786	D-dimer levels	1	0.5361446386000000	0.0091466924000000	15	293	53614
846	Response to antipsychotic treatment in schizophrenia (reasoning)	1	0.5391946081000000	0.0117524043000000	10	69	53919
904	Platelet aggregation	1	0.5440945591000000	0.0087065949000000	14	176	54409
40	Body mass index	1	0.5442245578000000	0.0011535093000000	120	2473	54422
328	Attention deficit hyperactivity disorder	1	0.5498245018000000	0.0016712561000000	73	1189	54982

669	Intelligence	1	0.5501344987000000	0.0125161043000000	9	113	55013
606	Estradiol levels	1	0.5508244918000000	0.0143938783000000	8	94	55082
626	Subcutaneous adipose tissue	1	0.5508344917000000	0.0033008476000000	37	335	55083
449	Thiazide-induced adverse metabolic effects in hypertensive patients	1	0.5513044870000000	0.0021163625000000	59	845	55130
411	PR interval	1	0.5516544835000000	0.0059152358000000	22	445	55165
1017	Protein quantitative trait loci	1	0.5530744693000000	0.0025255931000000	48	535	55307
997	Inattentive symptoms	1	0.5535644644000000	0.0090210816000000	15	177	55356
819	Cardiac hypertrophy	1	0.5541544585000000	0.0062602606000000	19	282	55415
593	Adiposity	1	0.5547644524000000	0.0152319547000000	8	250	55476
44	Parkinson's disease	1	0.5564244358000000	0.0017044134000000	70	3342	55642
59	Conotruncal heart defects	1	0.5580444196000000	0.0104810005000000	11	105	55804
438	Metabolite levels	1	0.5585544145000000	0.0014445903000000	87	1787	55855
171	Ejection fraction in Tripanosoma cruzi seropositivity	1	0.5616343837000000	0.0041183317000000	25	252	56163
136	Bone properties (heel)	1	0.5663043370000000	0.0124048115000000	11	297	56630
321	DNA methylation (variation)	1	0.5666743333000000	0.0064305824000000	19	386	56667
662	Response to Vitamin E supplementation	1	0.5710642894000000	0.0114949135000000	7	11	57106
125	Chronic obstructive pulmonary disease (moderate to severe)	1	0.5717642824000000	0.0131557132000000	9	234	57176
870	Radiation response	1	0.5734242658000000	0.0116404930000000	11	90	57342
238	Adverse response to chemotherapy in breast cancer (alopecia) (anti-microtubule)	1	0.5743342567000000	0.0156224382000000	7	86	57433
292	Atopic dermatitis	1	0.5754242458000000	0.0043553347000000	28	401	57542
432	Endometriosis	1	0.5765842342000000	0.0049005068000000	25	310	57658
273	Periodontitis (DPAL)	1	0.5787242128000000	0.0078614186000000	14	100	57872
854	HIV-1 control	1	0.5815741843000000	0.0024920817000000	46	758	58157
500	Response to antipsychotic treatment	1	0.5826041740000000	0.0024396185000000	55	1173	58260
368	Adverse response to chemotherapy (neutropenia/leucopenia) (paclitaxel + carboplatin)	1	0.5839841602000000	0.0144592208000000	7	109	58398
671	Vitamin B12 levels	1	0.5858741413000000	0.0140275283000000	7	170	58587
932	Red blood cell count	1	0.5864141359000000	0.0128064820000000	8	63	58641
173	Parasitemia in Tripanosoma cruzi seropositivity	1	0.5876041240000000	0.0046551168000000	24	457	58760
481	Tumor biomarkers	1	0.5915240848000000	0.0113742599000000	9	140	59152
224	Anxiety in major depressive disorder	1	0.5934140659000000	0.0104473220000000	8	49	59341
76	Thyroid peroxidase antibody positivity	1	0.5941740583000000	0.0113125923000000	11	257	59417
613	Immune reponse to smallpox (secreted IFN-alpha)	1	0.5954040460000000	0.0034638875000000	31	378	59540
696	IgA nephropathy	1	0.5984740153000000	0.0109355836000000	9	78	59847
468	Metabolite levels (5-HIAA)	1	0.5987040130000000	0.0070369042000000	13	62	59870
688	Hemostatic factors and hematological phenotypes	1	0.6002339977000000	0.0050896097000000	25	536	60023
239	Adverse response to chemotherapy in breast cancer (alopecia) (cyclophosphamide+doxorubicin+/-5f	1	0.6025239748000000	0.0137014435000000	8	58	60252
194	Fasting insulin (interaction)	1	0.6068439316000000	0.0087662623000000	14	189	60684
1	Response to serotonin reuptake inhibitors in major depressive disorder (plasma drug and metabolite	1	0.6073639264000000	0.0089197110000000	12	193	60736
923	Eosinophilic esophagitis (pediatric)	1	0.6093439066000000	0.0081799356000000	14	127	60934
574	Renal function-related traits (BUN)	1	0.6101538985000000	0.0091259607000000	15	233	61015
404	Ovarian cancer	1	0.6123038770000000	0.0081130626000000	14	527	61230
762	Progressive supranuclear palsy	1	0.6134238658000000	0.0104658544000000	11	1273	61342
989	Anthropometric traits	1	0.6150938491000000	0.0117739509000000	10	160	61509
285	Glycemic traits (pregnancy)	1	0.6170938291000000	0.0180424688000000	7	74	61709
121	Anorexia nervosa	1	0.6209737903000000	0.0036266360000000	26	142	62097
951	Methotrexate pharmacokinetics (acute lymphoblastic leukemia)	1	0.6227337727000000	0.0047980274000000	21	303	62273
317	Reading and spelling	1	0.6233837662000000	0.0129199972000000	10	81	62338
1006	Conduct disorder (interaction)	1	0.6242037580000000	0.0068544413000000	15	163	62420
73	Colorectal cancer (diet interaction)	1	0.6249837502000000	0.0079092839000000	13	331	62498
25	Acne (severe)	1	0.6256937431000000	0.0057771573000000	17	266	62569
635	Economic and political preferences (time)	1	0.6272537275000000	0.0115250446000000	9	60	62725
196	Homeostasis model assessment of insulin resistance (interaction)	1	0.6289237108000000	0.0090092937000000	15	204	62892
156	Chronic lymphocytic leukemia	1	0.6392136079000000	0.0033033199000000	36	624	63921
560	Glaucoma (primary open-angle)	1	0.6399936001000000	0.0112562438000000	10	109	63999
172	PR interval in Tripanosoma cruzi seropositivity	1	0.6504934951000000	0.0008555317000000	101	720	65049
93	Smoking initiation	1	0.6511134889000000	0.0038879581000000	27	508	65111
961	AIDS	1	0.6534734653000000	0.0147963371000000	7	161	65347
195	Homeostasis model assessment of beta-cell function (interaction)	1	0.6546534535000000	0.0081646350000000	12	115	65465
702	Fat distribution (HIV)	1	0.6551934481000000	0.0069250203000000	14	124	65519
426	Multiple myeloma (hyperdiploidy)	1	0.6566034340000000	0.0075450372000000	14	674	65660
274	Periodontitis (Mean PAL)	1	0.6583934161000000	0.0014998306000000	35	97	65839
56	Blood metabolite ratios	1	0.6605633944000000	0.0025049604000000	50	1227	66056
958	Bone mineral density (spine)	1	0.6632133679000000	0.0042736725000000	26	565	66321
911	Mortality in heart failure	1	0.6633233668000000	0.0087520887000000	11	299	66332
899	Osteoporosis-related phenotypes	1	0.6681833182000000	0.0116200371000000	8	124	66818
655	Response to tocilizumab in rheumatoid arthritis	1	0.6736832632000000	0.0038676962000000	28	569	67368
63	Response to inhaled corticosteroid treatment in asthma (percentage change of FEV1)	1	0.6749332507000000	0.0098799297000000	9	211	67493
131	Response to cytadine analogues (cytosine arabinoside)	1	0.6760232398000000	0.0045305236000000	22	186	67602
249	Lung function (forced vital capacity)	1	0.6767232328000000	0.0111851672000000	9	148	67672
990	Biochemical measures	1	0.6827331727000000	0.0068024632000000	16	208	68273
705	Ovarian reserve	1	0.6836031640000000	0.0156724113000000	7	102	68360
375	Dietary macronutrient intake	1	0.6847031530000000	0.0111140258000000	8	98	68470
712	Response to antidepressant treatment	1	0.6849631504000000	0.0042806706000000	22	698	68496
311	Systemic lupus erythematosus and Systemic sclerosis	1	0.6890031100000000	0.0063798772000000	20	454	68900
219	Liver enzyme levels (aspartate transaminase)	1	0.6908730913000000	0.0109019195000000	8	72	69087
721	Alzheimer's disease (age of onset)	1	0.6917330827000000	0.0077061375000000	12	150	69173
842	Total ventricular volume	1	0.6954830452000000	0.0053834086000000	19	163	69548
759	White blood cell types	1	0.6991330087000000	0.0050361777000000	19	184	69913
756	Dupuytren's disease	1	0.7011729883000000	0.0085616926000000	13	299	70117
451	Retinopathy in non-diabetics	1	0.7016529835000000	0.0076402129000000	11	107	70165
147	Glomerular filtration rate	1	0.7055829442000000	0.0107190498000000	8	133	70558
1005	Attention deficit hyperactivity disorder symptoms (interaction)	1	0.7057229428000000	0.0116842636000000	7	129	70572
782	Alcoholism (heaviness of drinking)	1	0.7127528725000000	0.0110089835000000	8	56	71275
758	Sudden cardiac arrest	1	0.7168628314000000	0.0018892580000000	50	554	71686
1003	Attention deficit hyperactivity disorder and conduct disorder	1	0.7181828182000000	0.0036281801000000	26	184	71818
118	Plasma amyloid beta peptide concentrations (ABx-40)	1	0.7192528075000000	0.0102812888000000	10	192	71925
19	Chronic hepatitis B infection	1	0.7221727783000000	0.0118892828000000	8	543	72217
148	Periodontal disease-related phenotypes	1	0.7289027110000000	0.0090243286000000	9	48	72890
599	Activated partial thromboplastin time	1	0.7349826502000000	0.0094090762000000	9	126	73498
673	Temperament (bipolar disorder)	1	0.7353826462000000	0.0078973875000000	13	444	73538
621	Response to antidepressants	1	0.7360326397000000	0.0094133483000000	8	84	73603
245	Serum protein levels (sST2)	1	0.7407125929000000	0.0063097016000000	14	190	74071
952	Hair morphology	1	0.7415025850000000	0.0062964958000000	15	144	74150
1015	Black vs. blond hair color	1	0.7450425496000000	0.0072186147000000	7	26	74504
993	Panic disorder	1	0.7475925241000000	0.0056419145000000	17	219	74759
65	Alzheimer's disease	1	0.7484925151000000	0.0016110759000000	68	1104	74849
309	Multiple sclerosis (OCB status)	1	0.7537024630000000	0.0070590975000000	10	140	75370
102	Bipolar disorder	1	0.7551124489000000	0.0010736960000000	109	1494	75511
944	Carotid atherosclerosis in HIV infection	1	0.7564424356000000	0.0098516928000000	7	109	75644
174	QRS duration in Tripanosoma cruzi seropositivity	1	0.7576024240000000	0.0024359160000000	38	433	75760
207	Barrett's esophagus	1	0.7591424086000000	0.0073121401000000	12	210	75914
60	Telomere length	1	0.7599224008000000	0.0041278027000000	26	752	75992
123	Serum uric acid levels	1	0.7602423976000000	0.0113047144000000	7	113	76024
619	Immune response to smallpox vaccine (IL-6)	1	0.7633423666000000	0.0023286122000000	44	710	76334
231	Systolic blood pressure in sickle cell anemia	1	0.7642523575000000	0.0011321798000000	10	49	76425
647	Fasting plasma glucose	1	0.7646023540000000	0.0085870414000000	11	126	76460
406	Hair color	1	0.7657823422000000	0.0058458348000000	13	146	76578
363	Adverse response to chemotherapy (neutropenia/leucopenia) (docetaxel)	1	0.7664523355000000	0.0094343488000000	7	200	76645
946	Major depressive disorder (broad)	1	0.7686923131000000	0.0095102631000000	9	44	76869
409	Tanning	1	0.7720022800000000	0.0043145687000000	15	198	77200
294	Coronary artery calcification	1	0.7741722583000000	0.0019493684000000	55	1114	77417
616	Immune reponse to smallpox (secreted IL-1beta)	1	0.7756222438000000	0.0050309429000000	11	114	77562
192	Blood pressure measurement (high sodium intervention)	1	0.7784022160000000	0.0061862048000000	7	25	77840
334	Post-traumatic stress disorder (asjusted for relatedness)	1	0.7808321917000000	0.0046654759000000	15	96	78083
26	Lupus nephritis in systemic lupus erythematosus	1	0.7809221908000000	0.0061587951000000	17	484	78092
852	Cannabis dependence	1	0.7829221708000000	0.0075593871000000	10	95	78292
863	Weight	1	0.7864321357000000	0.0033995820000000	33	849	78643
575	Renal function-related traits (eGRFcrea)	1	0.7867321327000000	0.0085967086000000	10	120	78673
752	Aging (time to event)	1	0.7872621274000000	0.0078412745000000	8	220	78726
667	Adverse response to lamotrigine and phenytoin	1	0.7874521255000000	0.0073056886000000	12	181	78745
469	Metabolite levels (5-HIAA/ MHPG Ratio)	1	0.7887721123000000	0.0056506784000000	11	85	78877
539	Smoking behavior	1	0.7900820992000000	0.0024892486000000	39	965	79008
934	Multiple sclerosis (severity)	1	0.7975520245000000	0.0078824550000000	11	137	79755
841	MRI atrophy measures	1	0.7996820032000000	0.0059573475000000	13	138	79968
483	Behcet's disease	1	0.8008519915000000	0.0083698898000000	12	166	80085
153	Bipolar disorder (body mass index interaction)	1	0.8031419686000000	0.0015610769000000	38	243	80314
726	Response to antipsychotic therapy (extrapyramidal side effects)	1	0.8046719533000000	0.0041646631000000	18	175	80467
389	Bulimia nervosa	1	0.8062219378000000	0.0031523317000000	23	223	80622
325	Sleep duration	1	0.8064319357000000	0.0038772268000000	7	23	80643
222	Calcium levels	1	0.8127018730000000	0.0043586766000000	20	445	81270
706	Response to hepatitis C treatment	1	0.8142118579000000	0.0056979265000000	14	150	81421

806	Metabolic syndrome (bivariate traits)	1	0.8238017620000000	0.0062460727000000	12	184	82380
24	Forced vital capacity	1	0.8242617574000000	0.0088934669000000	7	79	82426
992	Response to treatment for acute lymphoblastic leukemia	1	0.8253617464000000	0.0066345494000000	12	155	82536
248	Lung function (forced expiratory volume in 1 second)	1	0.8263117369000000	0.0083351352000000	9	95	82631
608	Male-pattern baldness	1	0.8268217318000000	0.0073679336000000	10	1525	82682
893	Conduct disorder	1	0.8277917221000000	0.0068171901000000	11	445	82779
170	Chagas cardiomyopathy in Tripanosoma cruzi seropositivity	1	0.8300616994000000	0.0038927210000000	18	118	83006
168	Large artery stroke	1	0.8306416936000000	0.0070329346000000	9	107	83064
909	Heart failure	1	0.8318016820000000	0.0054858461000000	17	247	83180
634	Economic and political preferences (immigration/crime)	1	0.8325216748000000	0.0046081800000000	12	89	83252
284	Personality dimensions	1	0.8340916591000000	0.0031117923000000	28	429	83409
622	Temperament	1	0.8372016280000000	0.0062157408000000	16	306	83720
135	Amyotrophic lateral sclerosis (sporadic)	1	0.8394616054000000	0.0010518895000000	95	1687	83946
261	Cocaine dependence	1	0.8397616024000000	0.0049084765000000	9	168	83976
326	Sleep quality	1	0.8400515995000000	0.0075611908000000	7	29	84005
540	Response to taxane treatment (docetaxel)	1	0.8422415776000000	0.0060893255000000	15	434	84224
55	Blood metabolite levels	1	0.8459715403000000	0.0007550241000000	153	3257	84597
586	Response to citalopram treatment	1	0.8493415066000000	0.0033461075000000	17	206	84934
583	Aging	1	0.8502914971000000	0.0062794617000000	9	205	85029
356	Adverse response to chemotherapy (neutropenia/leucopenia) (all antimicrotubule drugs)	1	0.8508814912000000	0.0064924144000000	12	175	85088
307	Migraine without aura	1	0.8513914861000000	0.0029299051000000	32	677	85139
986	Quantitative traits	1	0.8524114759000000	0.0028261716000000	28	338	85241
884	Self-rated health	1	0.8541314587000000	0.0071868565000000	10	63	85413
969	RR interval (heart rate)	1	0.8547114529000000	0.0042696065000000	19	351	85471
50	Plasma omega-6 polyunsaturated fatty acid levels (gamma-linolenic acid)	1	0.8559614404000000	0.0073568898000000	9	237	85596
251	Response to mTOR inhibitor (everolimus)	1	0.8574614254000000	0.0061831823000000	8	48	85746
748	vWF and FVIII levels	1	0.8593714063000000	0.0039245916000000	11	95	85937
837	Information processing speed	1	0.8645113549000000	0.0031137475000000	25	431	86451
213	Endometrial cancer	1	0.8672613274000000	0.0063562943000000	9	159	86726
405	Eye color	1	0.8701912981000000	0.0040190165000000	13	59	87019
333	Blood trace element (Zn levels)	1	0.8709212908000000	0.0046064051000000	11	151	87092
64	Response to radiotherapy in cancer (late toxicity)	1	0.8737912621000000	0.0079633432000000	9	82	87379
512	Body mass index (interaction)	1	0.8755712443000000	0.0047745129000000	12	126	87557
692	Prostate cancer (gene x gene interaction)	1	0.8765112349000000	0.0020035687000000	50	1065	87651
189	Inflammatory biomarkers	1	0.8770512295000000	0.0025148996000000	29	616	87705
338	Malaria	1	0.8772812272000000	0.0049963922000000	13	433	87728
1000	Multiple sclerosis (age of onset)	1	0.8779612204000000	0.0066290985000000	8	159	87796
728	Obesity (extreme)	1	0.8832811672000000	0.0045077346000000	17	439	88328
738	Metabolic traits	1	0.8879811202000000	0.0022494639000000	47	1192	88798
440	Myopia (pathological)	1	0.8901410986000000	0.0012016340000000	75	1027	89014
1032	Hip geometry	1	0.8915910841000000	0.0057159647000000	16	444	89159
789	Dehydroepiandrosterone sulphate levels	1	0.8920910791000000	0.0085464781000000	8	221	89209
361	Adverse response to chemotherapy (neutropenia/leucopenia) (cisplatin)	1	0.8957610424000000	0.0068084382000000	7	183	89576
611	Response to statin therapy	1	0.8963510365000000	0.0024174051000000	37	573	89635
590	Periodontal microbiota	1	0.8973610264000000	0.0036523483000000	15	190	89736
198	Hepatitis B	1	0.8984210158000000	0.0091426083000000	7	668	89842
81	Response to methotrexate in juvenile idiopathic arthritis	1	0.9014509855000000	0.0042656537000000	15	122	90145
113	Response to anti-retroviral therapy (ddI/d4T) in HIV-1 infection (Grade 1 peripheral neuropathy)	1	0.9028609714000000	0.0031951343000000	11	116	90286
628	Visceral adipose tissue/subcutaneous adipose tissue ratio	1	0.9034309657000000	0.0019949774000000	39	420	90343
627	Visceral adipose tissue adjusted for BMI	1	0.9055209448000000	0.0014621663000000	44	264	90552
176	Schizophrenia	1	0.9055409446000000	0.0008494828000000	130	2712	90554
91	Age of smoking initiation	1	0.9093809062000000	0.0075466937000000	8	139	90938
304	Migraine	1	0.9112608874000000	0.0013543605000000	69	1260	91126
281	Axial length	1	0.9118008820000000	0.0016665564000000	21	157	91180
506	Obesity-related traits	1	0.9132908671000000	0.0001244390000000	829	11120	91329
20	Age-related hearing impairment	1	0.9136308637000000	0.0032483947000000	27	850	91363
315	Corneal astigmatism	1	0.9238607614000000	0.0025401531000000	30	746	92386
1009	Response to TNF antagonist treatment	1	0.9257007430000000	0.0055654336000000	8	144	92570
393	Obesity (early onset extreme)	1	0.9275907241000000	0.0040311794000000	19	483	92759
38	Systemic lupus erythematosus	1	0.9287007130000000	0.0010537534000000	100	2777	92870
474	Metabolite levels (MHPG)	1	0.9349906501000000	0.0005194699000000	20	111	93499
894	Conduct disorder (symptom count)	1	0.9371906281000000	0.0042628478000000	15	503	93719
318	Word reading	1	0.9394206058000000	0.0041051443000000	14	135	93942
265	Waist circumference	1	0.9411605884000000	0.0023002171000000	24	469	94116
504	Alcohol and nictotine co-dependence	1	0.9418305817000000	0.0058215156000000	9	125	94183
112	Social communication problems	1	0.9425005750000000	0.0027006754000000	21	257	94250
513	Waist-to-hip circumference ratio (interaction)	1	0.9436005640000000	0.0037961630000000	14	250	94360
505	Non-alcoholic fatty liver disease histology (other)	1	0.9456905431000000	0.0006306330000000	20	156	94569
711	Depression and alcohol dependence	1	0.9466805332000000	0.0016285682000000	8	24	94668
299	Asthma (childhood onset)	1	0.9501704983000000	0.0025861866000000	25	1005	95017
651	Brain structure	1	0.9506204938000000	0.0023045951000000	21	254	95062
591	Breast size	1	0.9509004910000000	0.0016704644000000	31	379	95090
1040	Cognitive test performance	1	0.9519404806000000	0.0041759037000000	14	358	95194
499	Cardiac Troponin-T levels	1	0.9530604694000000	0.0033014382000000	17	215	95306
277	Metabolite levels (X-11787)	1	0.9531304687000000	0.0024758905000000	29	695	95313
508	Asthma	1	0.9538004620000000	0.0014231094000000	49	926	95380
895	Common traits (Other)	1	0.9578804212000000	0.0026748840000000	16	259	95788
554	Epilepsy (generalized)	1	0.9594904051000000	0.0049312159000000	10	258	95949
466	Lymphoma	1	0.9596304037000000	0.0039840316000000	9	261	95963
390	Eating disorders	1	0.9617503825000000	0.0027753585000000	15	334	96175
693	Capecitabine sensitivity	1	0.9631003690000000	0.0026396294000000	18	368	96310
460	Palmitic acid (16:0) plasma levels	1	0.9637103629000000	0.0028699199000000	12	154	96371
629	Visceral fat	1	0.9647303527000000	0.0017799982000000	39	532	96473
602	Bipolar disorder and schizophrenia	1	0.9676303237000000	0.0007192812000000	121	2188	96763
29	Alcohol dependence (age at onset)	1	0.9679403206000000	0.0019608599000000	26	499	96794
401	Attention deficit hyperactivity disorder (inattention symptoms)	1	0.9694203058000000	0.0024386760000000	7	73	96942
725	Cytomegalovirus antibody response	1	0.9703402966000000	0.0008986869000000	9	105	97034
175	QT interval in Tripanosoma cruzi seropositivity	1	0.9704302957000000	0.0019264374000000	14	136	97043
631	Economic and political preferences (environmentalism)	1	0.9715402846000000	0.0050252164000000	9	536	97154
331	Blood trace element (Cu levels)	1	0.9731802682000000	0.0045396357000000	7	345	97318
453	IgG glycosylation	1	0.9750502495000000	0.0002482819000000	368	5216	97505
515	Pancreatic cancer	1	0.9758602414000000	0.0018188105000000	38	547	97586
630	Economic and political preferences	1	0.9764902351000000	0.0032496485000000	17	254	97649
568	Orofacial clefts	1	0.9817501825000000	0.0014049079000000	44	555	98175
57	Immune response to measles-mumps-rubella vaccine	1	0.9820501795000000	0.0037945329000000	10	229	98205
501	Neuroticism	1	0.9824701753000000	0.0043830059000000	8	292	98247
289	Bronchopulmonary dysplasia	1	0.9827201728000000	0.0014689211000000	25	439	98272
559	Response to amphetamines	1	0.9828801712000000	0.0006445425000000	86	901	98288
1035	Aging traits	1	0.9830101699000000	0.0037863146000000	9	299	98301
433	QT interval (interaction)	1	0.9876301237000000	0.0014075365000000	17	195	98763
301	C-reactive protein	1	0.9882901171000000	0.0014314747000000	36	439	98829
933	Cognitive performance	1	0.9884801152000000	0.0006130797000000	117	1632	98848
180	Autism	1	0.9892001080000000	0.0014026165000000	12	130	98920
700	HIV-1 viral setpoint	1	0.9909800902000000	0.0018275728000000	24	634	99098
996	Hyperactive-impulsive symptoms	1	0.9911100889000000	0.0003851716000000	13	81	99111
94	Smoking quantity	1	0.9915500845000000	0.0021408825000000	8	86	99155
263	Epilepsy (remission after treatment)	1	0.9919700803000000	0.0021912318000000	7	109	99197
1042	Echocardiographic traits	1	0.9925200748000000	0.0018313208000000	8	189	99252
553	Amyotrophic lateral sclerosis (age of onset)	1	0.9932300677000000	0.0004588146000000	13	127	99323
101	Cognitive function	1	0.9962600374000000	0.0014952799000000	27	381	99626
380	Bilirubin levels	1	0.9975100249000000	0.0009566541000000	36	559	99751
275	Periodontitis (PAL4Q3)	1	0.9983600164000000	0.0004764082000000	14	216	99836
1029	Select biomarker traits	1	0.9984800152000000	0.0015263919000000	21	343	99848
319	Warfarin maintenance dose	1	0.9999800002000000	0.0005433861000000	9	531	99998
632	Economic and political preferences (fairness)	1	1	0	7	118	-1
878	Emphysema-related traits	1	1	0	7	95	-1
832	Serum prostate-specific antigen levels	1	1	0	7	120	-1
27	Hodgkin's lymphoma	1	1	0	16	165	-1
